# Risk of Importing Zoonotic Diseases through Wildlife Trade, United States

**DOI:** 10.3201/eid1511.090467

**Published:** 2009-11

**Authors:** Boris I. Pavlin, Lisa M. Schloegel, Peter Daszak

**Affiliations:** World Health Organization, Palikir, Federated States of Micronesia (B.I. Pavlin); Wildlife Trust, New York, New York, USA (L.M. Schloegel, P. Daszak)

**Keywords:** Zoonoses, communicable disease control, outbreaks, reservoirs, infectious disease transmission, RNA virus infections, hemorrhagic fevers, viruses, perspective

## Abstract

The United States is the world’s largest wildlife importer, and imported wild animals represent a potential source of zoonotic pathogens. Using data on mammals imported during 2000–2005, we assessed their potential to host 27 selected risk zoonoses and created a risk assessment that could inform policy making for wildlife importation and zoonotic disease surveillance. A total of 246,772 mammals in 190 genera (68 families) were imported. The most widespread agents of risk zoonoses were rabies virus (in 78 genera of mammals), *Bacillus anthracis* (57), *Mycobacterium tuberculosis* complex (48), *Echinococcus* spp. (41), and *Leptospira* spp. (35). Genera capable of harboring the greatest number of risk zoonoses were *Canis* and *Felis* (14 each), *Rattus* (13), *Equus* (11), and *Macaca* and *Lepus* (10 each). These findings demonstrate the myriad opportunities for zoonotic pathogens to be imported and suggest that, to ensure public safety, immediate proactive changes are needed at multiple levels.

## Abstract 2

Proactive changes are needed to protect the public from myriad serious diseases.

Most emerging infectious diseases are caused by zoonotic pathogens (*1*,*2*). The number and proportion of these diseases that originate in wild animals in particular has increased substantially in the past few decades, even after accounting for increased reports of new emerging infectious diseases (*1*). This trend and recent pandemics of wildlife-origin infectious diseases (e.g., HIV, severe acute respiratory syndrome) suggest that targeted surveillance efforts should focus on activities that bring humans and wildlife in close contact (*1*,*3*).

The United States is among the world’s largest importers of live wild animals (*4*) and imported >1 billion individual animals during 2000–2004 (*5*). Little disease surveillance is conducted for imported animals; quarantine is required for only wild birds, primates, and some ungulates arriving in the United States, and mandatory testing exists for only a few diseases (psittacosis, foot and mouth disease, Newcastle disease, avian influenza). Other animals are typically only screened for physical signs of disease, and pathogen testing is delegated to either the US Department of Agriculture (for livestock) or the importer (*6*). The process of preimport housing and importation often involves keeping animals at high density and in unnatural groupings of species, providing opportunities for cross-species transmission and amplification of known and unknown pathogens. Thus, imported wildlife remain a major public health threat, as exemplified by the importation of Ebola virus in primates from the Philippines (*7*), monkeypox from imported African rodents (*8*), and possibly HIV from chimpanzees in central Africa (*9*). Wildlife importation also poses a great threat to domestic wildlife and the US agriculture industry (*5*).

To analyze the volume and diversity of live mammals that have been imported into the United States in recent years, we used data from the US Fish and Wildlife Service Law Enforcement Management Information System. We focused on mammals because of the frequency and severity of previously reported mammal-borne zoonoses and because of the frequent close association between humans and many mammalian species (e.g., as pets). We then assessed the zoonotic diseases that imported mammals are known to host. Our results may be used to inform policy decisions about wildlife importation and zoonotic disease surveillance and may alert clinicians to the broad range of possible zoonoses that may be encountered in patients who have been exposed to imported animals.

## Methods

We used Freedom of Information Act requests to obtain records from the database of the US Fish and Wildlife Service Law Enforcement Management Information System. We obtained records for all wildlife shipments into the United States during 2000–2005 through 14 of the 18 designated animal importation ports (Anchorage, Alaska; Atlanta, Georgia; Baltimore, Maryland; Boston, Massachusetts; Chicago, Illinois; Dallas, Texas; Honolulu, Hawaii; Los Angeles, California; Miami, Florida; New Orleans, Louisiana; New York, New York; Portland, Oregon; San Francisco, California; and Seattle, Washington). Data were not available for Houston, Texas; Louisville, Kentucky; Memphis, Tennessee; and Newark, New Jersey. For each importation, we acquired information on the taxonomy, quantity, source (e.g., wild-caught, farmed), country of origin, intermediate port of call, port of entry, and declared purpose of all live specimens. Descriptive analyses were performed to determine the volume of trade from various regions of the world and the types of mammals imported. Individual importation events were then grouped into genera to determine the diversity of taxa imported. The phylogenetic relationships and geographic ranges of host mammals were determined by using the Animal Diversity Web at the University of Michigan Museum of Zoology (http://animaldiversity.ummz.umich.edu/site/index.html).

We searched the literature to identify the zoonotic pathogens known to occur in animals of each taxon in the database. Only data on live animal importations (as opposed to animal products) and importations for which the genus was known were retained for analysis. Statistical analyses were performed by using Intercooled Stata 9 (StataCorp, College Station, TX, USA). In our final risk assessment, we did not account for the origin of each specific importation because of limitations in the database, likely caused by a complicated system of exportation and reimportation.

We created a list of relevant zoonotic diseases at risk for importation (hereafter referred to as risk zoonoses) by searching the Centers for Disease Control and Prevention website (www.cdc.gov) and the World Health Organization website (www.who.int), reviewing the list of Select Agents (agents with bioterrorism potential) of the US Department of Health and Human Services (*10*), and consulting experts in the field. To be on the list, diseases had to meet the following 5 criteria: 1) the pathogen must be zoonotic (there must be a recorded instance of infection of a human from an animal source); 2) the pathogen must be capable of causing significant illness or death (e.g., fungal skin infections would not be on the list because although they are extremely common zoonoses, their effects are rarely debilitating); 3) the pathogen must be present in animals in the wild (i.e., not only in experimental models); 4) the pathogen must not currently be widespread in the United States, or it must have the potential for new epidemiology with regard to transmission (e.g., *Yersinia pestis* is presently found in wild rodents in the western United States, but it is not expected to be found in animals sold as pets); and 5) if the pathogen uses an intermediate vector, competent vectors must exist in the United States. The resulting list comprised 30 risk zoonoses (20 viral diseases, 9 bacterial diseases, and 1 helminthic disease); no fungal, protozoal, or prion diseases were on the list, and thus they were not analyzed.

Determination of the host range of the risk zoonoses was accomplished through systematic genus-driven and pathogen-driven searches of PubMed databases (www.pubmed.gov), the Google search engine (www.google.com), and references within published works. Confirmed presence was defined as either isolation of the pathogen from an animal or serologic evidence of past infection. For all animals identified in the literature as carrying a risk zoonosis, genus and family were recorded. The host ranges of all of the risk zoonoses were then cross-referenced against the imported genera to generate tables showing diseases found in each imported genus (affected genera). If the disease was found in a different genus within the same family, this was also noted (potentially affected genera). The justification for this expanded risk assessment is the host nonspecificity of many infectious diseases; lack of evidence for the presence of a given disease in a given host should not be construed as evidence against its presence.

## Results

During 2000–2005, a total of 4,067 shipment fractions of mammals were imported (a shipment fraction is the sum of all animals of a single species in a given shipment; a single shipment may contain several shipment fractions), totaling 246,772 individual mammals and representing 190 genera and 68 families. The average number of animals per shipment fraction was 61 (range 1–8,000). The most common declared purpose for importation was commercial use (not classified according to pet trade, food, traditional medicine, etc.), accounting for 66% (163,760 individuals) of the total. The second most common declared purpose was biomedical research, accounting for 28% (69,986 individuals) of the total. Only a small number of individuals were imported for breeding, educational, zoo, personal, and other uses. Numbers of the most commonly imported animals were 126,014 (>50% of all imported individuals) long-tailed macaques (*Macaca fascicularis*), 30,058 small desert hamsters (*Phodopus sungorus*), 19,724 rhesus macaques (*Macaca mulatta*), 19,537 raccoons (*Procyon lotor*), and 7,112 chinchillas (*Chinchilla lanigera*). Together, these 5 species accounted for 82% of all imported individuals. By number of shipment fractions, the most common animals were 1,343 *M. fascicularis* macaques, 332 *Callithrix jacchus* marmosets, 229 *M. mulatta* macaques, 165 *C. lanigera* chinchillas, and 107 *Potos flavus* kinkajous.

The most common countries of origin for animal shipment fractions were People’s Republic of China (717 shipment fractions); Guyana (635), United Kingdom (359), Vietnam (314), and Indonesia (305). These values must be interpreted cautiously, however, because many animals are imported and then reexported; thus, their true origin may become obscured. For example, a “wild-caught” chinchilla with a “country of origin” of Czech Republic must have originated elsewhere because chinchillas are native to Chile. A comparison between the natural geographic range of all wild-caught animals and their stated countries of origin showed that >25% of the pairings were impossible (i.e., the animals could not have come from their stated country of origin). This limitation is inherent in the way US Fish and Wildlife Service Law Enforcement Management Information System data are collected, and we were unable to correct these data.

The source of the animals was largely uninterpretable because 49% of all individuals were declared as being sourced from “animal derivatives and parts,” despite the fact that we had selected only live animals for our analysis, and despite the fact that “animal derivatives and parts” is not one of the permitted responses to this question. Another 29% were declared as “captive-bred” and 15% as “wild-caught.”

For the final list of risk zoonoses, 3 of the original 30 agents (Hendra virus, Menangle virus, and *Rickettsia prowazekii*) were removed because few, if any, genera were found to harbor these infections; the final tables therefore include 27 diseases (Tables 1–3). The risk zoonoses capable of infecting the greatest number of genera were: rabies viruses, in 78 genera; *Bacillus anthracis*, the causative agent of anthrax, in 57 genera; *Mycobacterium tuberculosis* complex, in 48 genera; *Echinococcus* spp., the agents of hydatid cyst disease, in 41 genera; *Leptospira* spp., in 35 genera; *Brucella* spp., the agents of undulant fever, in 32 genera; *Francisella tularensis*, the agent of tularemia, in 31 genera; Crimean-Congo hemorrhagic fever virus, in 27 genera; *Y. pestis*, the agent of plague, in 24 genera; and *Coxiella burnetii*, the agent of Q fever, in 20 genera (Table 2; Technical Appendix).

**Table 1:** Risk zoonoses and their associated clinical syndromes in humans*

Pathogen	Primary clinical syndrome in humans
Viruses	
Lymphocytic choriomeningitis virus	Aseptic meningitis
Cercopithecine herpesvirus-1 (herpes B)	Encephalitis
Nipah virus	Encephalitis
Rabies viruses†	Encephalitis
Venezuelan equine encephalitis virus	Encephalitis
Tick-borne encephalitis virus complex†	Encephalitis or hemorrhagic fever
Crimean-Congo hemorrhagic fever virus	Hemorrhagic fever
Ebola viruses†	Hemorrhagic fever
Lassa fever virus	Hemorrhagic fever
Marburg virus	Hemorrhagic fever
Rift Valley fever virus	Hemorrhagic fever
South American hemorrhagic fever arenaviruses†	Hemorrhagic fever
Hantaviruses associated with HFRS†	Hemorrhagic fever with nephropathy
Hantaviruses associated with HCPS†	Severe respiratory syndrome
Highly pathogenic avian influenza (H5N1) virus	Severe respiratory syndrome
SARS virus (or SARS-like CoV)	Severe respiratory syndrome
Yellow fever virus	Systemic illness or hemorrhagic fever
Monkeypox virus	Systemic illness or rash
Bacteria	
*Brucella* spp.	Systemic illness
*Coxiella burnetii*	Systemic illness
*Leptospira* spp.	Systemic illness
*Bacillus anthracis*	Varies by site of infection
*Burkholderia mallei*	Varies by site of infection
*Francisella tularensis*	Varies by site of infection
*Mycobacterium tuberculosis* complex†	Varies by site of infection
*Yersinia pestis*	Varies by site of infection
Helminths, *Echinococcus* spp.	Hydatid cyst disease

*Risk zoonoses, relevant zoonotic diseases at risk for importation into the United States; HFRS, hemorrhagic fever with renal syndrome; HCPS, hantavirus cardiopulmonary syndrome; SARS, severe acute respiratory syndrome; CoV, coronavirus. †Rabies viruses includes the zoonotic lyssaviruses Australian bat lyssavirus, Duvenhage, European bat lyssavirus 1 and 2, Mokolo, and rabies (*11*); tick-borne encephalitis complex includes Kyasanur Forest disease, Omsk hemorrhagic fever, and tickborne encephalitis (*11*); Ebolaviruses include Bundibugyo, Côte d'Ivoire, Reston, Sudan, and Zaire (*11*); epidemiologically relevant South American hemorrhagic fever arenaviruses include Guanarito, Junin, Machupo, and Sabia (*11*); hantaviruses associated with HFRS include Dobrava, Hantaan, Puumala, Saaremaa, and Seoul (*11*); hantaviruses associated with HCPS include Andes, Bayou, Black Creek Canal, Laguna Negra, New York, and Sin Nombre (*11*); *Mycobacterium tuberculosis* complex species are *M. africanum, M. bovis, M. bovis BCG, M. caprae, M. microti, M. pinnipedii,* and *M. tuberculosis hominis* (*12*).

**Table 3:** Mammal genera capable of harboring the greatest number of risk zoonoses*

Genus (common name)	No. (%) risk zoonoses
*Canis* (dogs)	14 (52)
*Felis* (cats)	14 (52)
*Rattus* (rats)	13 (48)
*Equus* (horses)	11 (41)
*Macaca* (macaques)	10 (37)
*Lepus* (rabbits and hares)	10 (37)
*Ovis* (sheep)	9 (33)
*Vulpes* (foxes)	9 (33)

*Risk zoonoses, relevant zoonotic diseases at risk for importation into the United States; n = 27.

**Table 2:** Risk zoonoses capable of infecting the greatest number of imported mammal genera

Pathogen	No. (%) affected genera*	No. (%) potentially affected genera†
Rabies viruses‡	78 (41)	155 (82)
*Bacillus anthracis*	57 (30)	113 (59)
*Mycobacterium tuberculosis* complex‡	48 (25)	124 (65)
*Echinococcus* spp.	41 (22)	89 (47)
*Leptospira* spp.	35 (18)	131 (69)
*Brucella* spp.	32 (17)	95 (50)
*Francisella tularensis*	31 (16)	115 (61)
Crimean-Congo hemorrhagic fever virus	27 (14)	91 (48)
*Yersinia pestis*	24 (13)	101 (53)
*Coxiella burnetii*	20 (11)	108 (57)

*Risk zoonosis (relevant zoonotic disease at risk for importation into the United States) identified in genus; n = 190. †Risk zoonosis identified in different genus within same family; n = 190. ‡Risk zoonoses, relevant zoonotic diseases at risk for importation into the United States. Refer to Table 1 footnote for explanation of pathogen complexes.

If each genus within affected families is counted as potentially capable of harboring a risk zoonosis (according to the principle that many diseases are not entirely host specific), the number of genera potentially capable of harboring rabies viruses rises to 155 (82% of all imported taxa); potential carriers of *Leptospira* spp. increase to 131; *M*. *tuberculosis* complex to 124; *F*. *tularensis* to 115; *B*. *anthracis* to 113; *C*. *burnetii* to 108; and *Y*. *pestis* to 101.

The genera capable of harboring the greatest number of risk zoonoses were *Canis* (dogs) and *Felis* (cats), 14 risk zoonoses each; *Rattus* (rats), 13; *Equus* (horses), 11; *Macaca* (macaques), 10; *Lepus* (rabbits and hares), 10; and *Ovis* (sheep) and *Vulpes* (foxes), 9 each (Table 3). Of the individuals in these high-risk genera, 49% were intended for commercial purposes and 44% were intended for biomedical research.

The families found to harbor the most risk zoonoses (excluding Hominidae because, by definition, they are capable of harboring all zoonotic diseases) were Muridae (Old World mice and rats, gerbils, whistling rats, and relatives), 21 risk zoonoses; Cricetidae (New World rats and mice, voles, hamsters, and relatives), 20; Canidae (coyotes, dogs, foxes, jackals, and wolves), 16; and Bovidae (antelopes, cattle, gazelles, goats, sheep, and relatives) and Felidae (cats), 15 each.

## Discussion

Our data demonstrate that myriad opportunities exist for key zoonotic pathogens to be imported into the United States or, if already present, to be introduced in a new context (e.g., in an animal sold as a pet). Imported animals of a large number of taxa were found to be capable of carrying risk zoonoses; these diseases include such serious public health threats as rabies, the filovirus hemorrhagic fevers, tuberculosis, and highly pathogenic avian influenza.

This study likely underestimates the broad nature of risk associated with the importation of wild animals. We examined only families in the class Mammalia that have been shown to harbor risk zoonoses; however, many pathogens routinely cross boundaries at least as high as the class level (e.g., human psittacosis from birds), if not higher. Furthermore, we included only live animals in this analysis; recent outbreaks associated with animal products (e.g., cutaneous anthrax from an imported goat hide used for making drums) attest to the risks associated even with dead animals (*13*). Finally, the study can neither estimate the risk for unknown pathogens, which may be imported but not yet identified, nor assess the volume and zoonotic risk created by illegal wildlife trade. Animals may be smuggled specifically because they have been banned from trade as a result of perceived or recognized health threats. Some animals on our list of risk zoonoses have already been banned from importation (e.g., masked palm civets, birds from countries affected by highly pathogenic avian influenza [H5N1]) (*14*). However, pathogens have been identified in illegally imported wildlife; e.g., a pair of crested hawk-eagles (*Spizaetus nipalensis*) smuggled from Thailand and recently confiscated in Belgium were infected with highly pathogenic avian influenza (H5N1) (*15*).

We did not quantitatively assess the risk for transmission of each pathogen at each importation event. Rather, we attempted to demonstrate the breadth of risk associated with importations of wild animals in general. Quantitative prevalence of the various pathogens in each wildlife host is highly variable, and determining it is beyond the scope of our analysis. Some genera represent the primary reservoirs of certain pathogens (e.g., *Peromyscus* for certain hantaviruses), whereas proof of the permissiveness of other genera to certain pathogens is limited to isolated case reports (e.g., Ebola Zaire virus in the duiker *Cephalophus*). Perhaps the greatest unknown associated with quantifying risks for each of the zoonoses is a pathogen’s infectivity in various hosts. Some pathogens may increase to a high enough load in their hosts to be infectious; others may cause nothing more than a measurable serologic response in what is otherwise a dead-end host (though explicitly known dead-end hosts have been excluded from these analyses).

Our analysis highlights several ways that the US Fish and Wildlife Service could improve data collection. To enhance public health officials’ ability to trace back the sources of imported zoonotic diseases, record keeping of the point of origin of shipments could be expanded to include not just their most recent and previous point of origin (as is currently done with the “Country of Origin” and “Country of Importation/Exportation/Re-importation”) but also their actual origin. Accurate recording of the source of the animals (e.g., wild-caught, captive-bred) is also needed. Our results showed that half of all individuals had a declared source that was not one of the allowed choices (e.g., wild-caught, captive-bred). The source of an animal affects not only the likely level of risk (i.e., one would expect captive-bred individuals to carry fewer zoonotic diseases than wild-caught individuals) but also mitigation strategies when zoonotic diseases are identified (e.g., euthanizing a colony vs. improving quarantine after capture).

The potential for importation of zoonoses that would pose a major public health threat suggests that increased surveillance should be applied to imported wildlife in the United States. One opportunity to reduce this threat is restriction of importation of key high-risk species, as was done when the Centers for Disease Control and Prevention used emergency powers to restrict importation of Gambian pouched rats during the monkeypox outbreak (*14*). Given the great diversity of animals identified by our analysis as potentially hazardous, broad importation bans would likely be necessary if the goal were to substantially decrease the overall risk. Political or social support may be limited for such broad bans, both in the United States (as one of the world’s largest purchasers of wildlife) and abroad (where wildlife trade can have profound economic benefits).

Furthermore, illegalizing trade may only increase underground (illicit) trade, thereby eliminating the possibility of screening shipments for potential hazards. A more effective and acceptable strategy would be enhancing surveillance for the specific pathogens noted for the key risk genera (those harboring the greatest number of risk zoonoses, i.e., *Canis, Felis*, *Rattus*, *Equus*, *Macaca*, *Lepus*, *Ovis,* and *Vulpes*). Notably, the numbers of shipments of mammals is low relative to other wildlife groups (e.g., fish and reptiles). Lawmakers’ interests in protecting our borders from external bioterrorism threats intersect with the need to protect ourselves from zoonotic diseases; many Category A bioterrorism threats (e.g., anthrax, plague, tularemia, and the viral hemorrhagic fevers) (*10*) are represented in the risk zoonoses outlined above. Finally, to facilitate the standardization of surveillance and detection of infection events, the Council of State and Territorial Epidemiologists should include all of the risk zoonoses among their states’ notifiable diseases (most of which are already included).

Perhaps one of the simplest practical interventions for minimizing zoonotic disease risk is reduction of opportunities for transmission from wildlife to humans. Although a large proportion of imported animals are destined for biomedical research (in which potential occupational risks are largely understood and quarantine procedures likely mitigate risk), a greater proportion (even among the high-risk genera) are destined for commercial use and therefore could expose a wider group of persons to zoonotic diseases. Education of professionals likely to come in close contact with imported animals (e.g., veterinarians, importers, pet store employees), as well as the general public, should emphasize the risks for contracting zoonotic diseases from wildlife and pets (*16*) and the need for proper hygiene, safety procedures, and personal protective equipment (*17*).

The recommendations above mirror others that exist in policy documents by the Defenders of Wildlife (*5*), in the 2003 joint position statement by the National Association of Public Health Veterinarians and the Council of State and Territorial Epidemiologists (*18*), and in a recent Policy Forum article (*19*). These reports describe clear steps for mitigating the risks presented by imported wildlife, yet their recommendations have so far gone largely unheeded. To ensure public safety, immediate proactive changes are needed at multiple levels. Such measures would be most effective if organized in consultation with groups involved in the wildlife trade.

## Supplementary Material

Technical Appendix Risk zoonoses capable of infecting mammals imported into the United States, 2000-2005
