# Supplementary material for: Risk of Importing Zoonotic Diseases through Wildlife Trade, United States
Source: Emerg Infect Dis. 2009 Nov;15(11):1721–6. doi: 10.3201/eid1511.090467 (PMC2857234; doi:10.3201/eid1511.090467)
Supplement: Technical Appendix — Risk zoonoses capable of infecting mammals imported into the United States, 2000-2005 [file 09-0467_Techapp-s1.pdf]

Risk of Importing Zoonotic Diseases through Wildlife Trade, United States

Technical Appendix

Technical Appendix Table. Risk zoonoses capable of infecting mammals imported into the United States, 2000–2005

Genus	Common name or type	Family	Helminths	Bacteria								Viruses																		Total Risk Zoonoses in genus	Total potential Risk Zoonoses (i.e., Risk Zoonosis found within family)
			Echinococcus spp. [2,80,163-184,186,196,203]	*Yersinia pestis* [2,32,80,101-105]	*Mycobacterium tuberculosis* complex* [2,49,80,116-135,196,200,202,217]	*Francisella tularensis* [2,80,150-162,188,201]	*Burkholderia mallei* [`;2,80,149]	*Bacillus anthracis* [2,49,80,84-93]	*Leptospira* spp. [2,80,136-148,187,202,205,209,210,218,219,232]	*Coxiella burnetii* [2,80,106-115,187,204,205,207,220]	*Brucella* spp. [2,49,80,94-100,208,211,221]	Monkeypox virus [2,80-82,198,199,206]	Yellow fever virus [2,20,80,202]	SARS virus (or SARS-like CoV) [2,31,80,194,225,226]	Highly pathogenic avian influenza (H5N1) virus [`;2,49,80,83,-215]	Hantaviruses associated with HCPS* [2,78-80,231]	Hantaviruses associated with HFRS* [2,78-80,190,191,216,231]	South American hemorrhagic fever arenaviruses* [2,3,16,17,80]	Rift Valley fever virus [1,2,7-15,80,190]	Marburg virus [2,28,29,80,212,225]	Lassa fever virus [2,49,80]	Ebola viruses* [1,2,24-28,80,189,223]	Crimean-Congo hemorrhagic fever virus [2-26,80]	Tick-borne encephalitis virus complex* [2,18,19,76,80,186,191,198]	Venezuelan equine encephalitis virus [1,2,21-23,80]	Rabies viruses* [2,32-74,80,135,195,227-229,233]	Nipah virus [2,77,80,230]	Cercopithecine herpesvirus-1 (herpes B) [2,75,80,192,193]	Lymphocytic choriomeningitis virus [2,29,30,80,222,226]		
Acinonyx	**Cheetah**	*Felidae*	o	o	X	o	o	X	o	o	o			o	o				o				o			X	o			3	15
Aepyceros	**Impala**	*Bovidae*	X	o	X	o	o	X	o	o	X								X			o	X	o	o	X				7	15
Ailuropoda	**Giant panda**	*Ursidae*		o		o		o	X	o	o															o				1	7
Alcelaphus	**Hartebeest**	*Bovidae*	o	o	o	o	o	X	o	o	X								o			o	X	o	o	o				3	15
Alopex	**Fox**	*Canidae*	X	o	o	X	o	o	o	o	X			o	o				o				o		o	X	o			4	16
Amblonyx	**Otter**	*Mustelidae*		o	o	o		o	o	o	o		o	o	o											o				-	11

Antidorcas	**Springbok**	*Bovidae*	**o**	**o**	**X**	**o**	**o**	**X**	**o**	**o**	**o**								**o**			**o**	**X**	**o**	**o**	**X**				**4**	**15**
Aonyx	**Clawless otter**	*Mustelidae*		**o**	**o**	**o**		**o**	**o**	**o**	**o**		**o**	**o**	**o**											**o**				**-**	**11**
Aotus	**Monkey**	*Aotidae*			**X**	**o**		**o**	**o**	**o**		**o**	**o**		**o**					**o**		**o**				**o**		**o**		**1**	**12**
Atelerix	**Hedgehog**	*Erinaceidae*			**o**	**X**			**o**			**X**											**X**			**X**				**4**	**6**
Bison	**Bison**	*Bovidae*	**X**	**o**	**X**	**o**	**o**	**X**	**o**	**o**	**X**								**o**			**o**	**o**	**o**	**o**	**X**				**5**	**15**
Cacajao	**Uakari**	*Cebidae*			**o**	**o**						**o**	**o**													**o**			**o**	**-**	**6**
Callithrix	**Monkey**	*Cebidae*			**X**	**X**						**o**	**X**													**X**			**X**	**5**	**6**
Callosciurus	**Squirrel**	*Sciuridae*	**o**	**o**		**o**			**o**	**o**		**o**							**o**				**o**	**o**		**o**				**-**	**10**
Canis	**Canine**	*Canidae*	**X**	**X**	**X**	**X**	**X**	**o**	**X**	**X**	**X**			**o**	**X**				**X**				**X**		**X**	**X**	**X**			**14**	**16**
Capromys	**Rodent**	*Capromyidae*																												**-**	**-**
Caracal	**Caracal**	*Felidae*	**o**	**o**	**o**	**o**	**o**	**X**	**o**	**o**	**o**			**o**	**o**				**o**				**o**			**X**	**o**			**2**	**15**
Castor	**Beaver**	*Castoridae*	**X**			**X**																				**X**				**3**	**3**
Cavia	**Guinea pig**	*Caviidae*		**X**				**X**																		**X**				**3**	**3**
Cebus	**Monkey**	*Cebidae*			**X**	**o**						**o**	**X**													**o**		**X**	**o**	**3**	**7**
Cephalophus	**Duiker antelope**	*Bovidae*	**o**	**o**	**o**	**o**	**o**	**o**	**o**	**o**	**o**								**o**			**X**	**o**	**o**	**o**	**o**				**1**	**15**
Cephalorhynchus	**Dolphin**	*Delphinidae*		**o**	**o**	**o**			**o**		**X**	**o**	**o**												**o**	**o**				**1**	**9**
Ceratotherium	**White rhinoceros**	*Rhinocerotidae*			**X**			**X**	**o**										**X**				**X**							**4**	**5**
Cercopithecus	**Guenon**	*Cercopithecidae*			**X**	**X**		**X**	**X**	**o**		**X**	**X**		**o**					**X**		**o**				**o**		**o**		**7**	**12**
Cerdocyon	**Fox**	*Canidae*	**X**	**o**	**o**	**o**	**o**	**o**	**o**	**o**	**o**			**o**	**o**				**o**				**o**		**o**	**X**	**o**			**2**	**16**
Cervus	**Deer**	*Cervidae*	**X**	**o**	**X**	**o**		**X**	**X**	**X**	**X**															**X**				**7**	**9**
Chaeropus	**Bandicoot**	*Peramelidae*																												**-**	**-**
Chaetophractus	**Armadillo**	*Dasypodidae*																								**o**				**-**	**1**
Cheirogaleus	**Dwarf lemur**	*Cheirogaleidae*																												**-**	**-**
Chinchilla	**Chincilla**	*Chinchillidae*										**X**														**X**				**2**	**2**

Chlorocebus	**Monkey**	*Cercopithecidae*			o	o		o	o	o		o	o		o					X		o				o		X		2	12
Choloepus	**Sloth**	*Megalonychidae*											X																	1	1
Civettictis	**African civet**	*Viverridae*			o			X	o					o	o								o			X				2	7
Coendou	**Porcupine**	*Erethizontidae*											X																	1	1
Colobus	**Monkey**	*Cercopithecidae*			o	o		X	o	o		o	X		o					o		o				X		X		4	12
Connochaetes	**Wildebeest**	*Bovidae*	X	o	X	o	o	X	o	o	X								X			o	o	o	o	o				5	15
Cricetomys	**Pouched rat**	*Nesomyidae*										X																		1	1
Cricetus	**Hamster**	*Cricetidae*	o	X	o	X		o	o	o	o	o		o	o	o	o	o	o		o		o	o		X			o	3	20
Crocuta	**Hyena**	*Hyaenidae*	X		X			X	X		X															X				6	6
Crossarchus	**Mongoose**	*Herpestidae*			o																					X				1	2
Cryptomys	**Mole rat**	*Bathyergidae*																												-	-
Cryptoprocta	**Fosa**	*Viverridae*			o			X	o					o	o								o			o				1	7
Cryptotis	**Shrew**	*Soricidae*				o											o					o		o		o				-	5
Cynictis	**Mongoose**	*Herpestidae*			o																					X				1	2
Cynomys	**Prairie dog**	*Sciuridae*	o	X		X			o	o		X							o				o	o		o			X	4	11
Dactylopsila	**Striped possum**	*Petauridae*							o																					-	1
Damaliscus	**Antelope**	*Bovidae*	X	o	X	o	o	X	o	o	X								o			o	X	o	o	X				6	15
Dasyprocta	**Agouti**	*Dasyproctidae*	X										X																	2	2
Dasypus	**Armadillo**	*Dasypodidae*																								X				1	1
Dendrohyrax	**Hyrax**	*Hyracoidea*		o				o																		X				1	3
Dendrolagus	**Kangaroo**	*Macropodidae*	o					o		o																				-	3
Desmodus	**Vampire bat**	*Phyllostomidae*																							X	X				2	2
Dolichotis	**Mara**	*Caviidae*		o				o																		o				-	3
Eira	**Tayra**	*Mustelidae*		o	o	o		o	o	o	o		X	o	o											o				1	11

Elephas	**Asian elephant**	*Elephantidae*			X			X															o			X				**3**	**4**
Eliomys	**Dormouse**	*Gliridae*										o					o													**-**	**2**
Equus	**Horse**	*Equidae*	X		X		X	X	X	X	X								X				X		X	X				**11**	**11**
Erinaceus	**Hedgehog**	*Erinaceidae*			X	X			X			o											o			o				**3**	**6**
Euoticus	**Galago**	*Galagidae*			o				o				o													o				**-**	**4**
Euphractus	**Armadillo**	*Dasypodidae*																								o				**-**	**1**
Eutamias	**Chipmunk**	*Sciuridae*	o	X		X			o	X		o							o				o	o		X				**4**	**10**
Felis	**Cat**	*Felidae*	X	X	X	X	X	X	X	X	o			X	X				X				X			X	X			**14**	**15**
Fossa	**Malagasy civet**	*Viverridae*			o			o	o					o	o								o			o				**-**	**7**
Funambulus	**Palm squirrel**	*Sciuridae*	o	o		o			o	o		o							o				o	X		o				**1**	**10**
Galago	**Galago**	*Galagidae*	X		X				X				X													X				**5**	**5**
Galictis	**Grison**	*Mustelidae*		o	o	o		X	o	o	o		o	o	o											o				**1**	**11**
Gazella	**Gazelle**	*Bovidae*	X	o	o	o	o	X	X	o	X								o			o	o	o	o	o				**4**	**15**
Genetta	**Genet**	*Viverridae*			X			X	X					o	o								X			X				**5**	**7**
Geomys	**Gopher**	*Geomyidae*																												**-**	**-**
Gerbillus	**Gerbil**	*Muridae*	o	o	o	o		o	o	o	o	o		o	o	o	o	o	o		o	o	o	o		X			o	**1**	**21**
Glossophaga	**Long-tongued bat**	*Phyllostomidae*																							o	X				**1**	**2**
Grammomys	**Thicket rat**	*Muridae*	o	X	o	o		o	o	o	o	o		o	o	o	o	o	o		o	o	o	o		o			o	**1**	**21**
Helarctos	**Sun bear**	*Ursidae*		o		o		o	o	o	o															o				**-**	**7**
Hemiechinus	**Indian hedgehog**	*Erinaceidae*			o	X			o			o											o			o				**1**	**6**
Hemitragus	**Tahr goat**	*Bovidae*	o	o	o	o	o	o	o	o	o								o			o	o	o	o	o				**-**	**15**
Herpestes	**Mongoose**	*Herpestidae*			o																					X				**1**	**2**
Heterocephalus	**Naked mole rat**	*Bathyergidae*																												**-**	**-**
Heterohyrax	**Hyrax**	*Procaviidae*		o				o																		o				**-**	**3**

Hexaprotodon	**Pygmy hippopotamus**	*Hippopotamidae*									o																			-	1
Hippotragus	**Antelope**	*Bovidae*	X	o	X	o	o	X	o	o	X								X			o	X	o	o	X				7	15
Hyaena	**Hyena**	*Hyaenidae*	X		o			o	o		o															X				2	6
Hydrochaeris	**Capybara**	*Hylobatidae*			o				X		X																			2	3
Hylobates	**Gibbon**	*Hylobatidae*			X				o		o	X																		2	4
Hylochoerus	**Forest hog**	*Suidae*	o		o			o	o		o			o	o		o					o	o		o	o				-	12
Hyperoodon	**Bottlenose whale**	*Ziphiidae*																												-	-
Hypogeomys	**Malagasy giant rat**	*Muridae*	o	o	o	o		o	o	o	o	o		o	o	o	o	o	o		o	o	o	o		o			o	-	21
Hystrix	**African porcupine**	*Hystricidae*						X															X			X				3	3
Lemniscomys	**Rodent**	*Muridae*	o	X	o	o		o	o	o	o	o		o	o	o	o	o	o		o	o	o	o		X			o	2	21
Leontopithecus	**Monkey**	*Cebidae*			o	X						o	o													o			X	2	6
Leptailurus	**Cat**	*Felidae*	o	o	o	o	o	o	X	o	o			o	o				o				o			o	o			1	15
Lepus	**Hare**	*Leporidae*	X	X	X	X			X	X	X	o											X	X		X				1-	11
Litocranius	**Antelope**	*Bovidae*	o	o	o	o	o	X	o	o	o								o			o	o	o	o	o				1	15
Lophocebus	**Mangabey**	*Cercopithecidae*			o	o		o	o	o		o	o		o					o		o				o		o		-	12
Loxodonta	**African elephant**	*Elephantidae*			X			X															X			X				4	4
Lutra	**Otter**	*Mustelidae*		o	o	o		o	o	o	X		o	o	o											X				2	11
Lycaon	**Wild dog**	*Canidae*	X	o	o	o	o	X	o	o	X			o	o				o				o		o	X	o			4	16
Lynx	**Cat**	*Felidae*	X	X	X	o	o	X	o	o	X			o	o				o				o			X	o			6	15
Macaca	**Monkey**	*Cercopithecidae*	X		X	X		o	X	X		X	X		X					o		X				o		X		1-	13
Macropus	**Kangaroo**	*Macropodidae*	X					X	X	X																				4	4
Macroscelides	**Elephant shrew**	*Macroscelididae*										o																		-	1
Madoqua	**Dik-dik antelope**	*Bovidae*	o	o	o	o	o	X	o	o	o								o			o	o	o	o	o				1	15
Manis	**Pangolin**	*Manidae*																												-	-

Marmosa	**Mouse opossum**	*Didelphidae*		**o**	**o**	**o**			**o**		**o**	**o**	**o**												**X**	**o**				**1**	**9**
Mastomys	**Multimammate rat**	*Muridae*	**X**	**o**	**o**	**o**		**o**	**X**	**o**	**o**	**o**		**o**	**o**	**o**	**o**	**o**	**o**		**X**	**o**	**X**	**o**		**X**			**o**	**5**	**21**
Mellivora	**Badger**	*Mustelidae*		**o**	**X**	**o**		**X**	**o**	**o**	**o**		**o**	**o**	**o**											**X**				**3**	**11**
Melursus	**Sloth bear**	*Ursidae*		**o**		**o**		**o**	**o**	**o**	**o**															**X**				**1**	**7**
Mephitis	**Skunk**	*Mustelidae*		**X**	**o**	**X**		**X**	**X**	**X**	**X**		**o**	**o**	**o**											**X**				**7**	**11**
Meriones	**Jird**	*Muridae*	**X**	**X**	**o**	**X**		**o**	**o**	**o**	**o**	**o**		**o**	**o**	**o**	**o**	**o**	**o**		**o**	**o**	**o**	**o**		**o**			**o**	**3**	**21**
Mesocricetus	**Hamster**	*Muridae*	**o**	**o**	**o**	**X**		**o**	**o**	**o**	**o**	**o**		**o**	**o**	**o**	**o**	**o**	**o**		**o**	**o**	**o**	**o**		**o**			**o**	**1**	**21**
Microcebus	**Mouse lemur**	*Cheirogaleidae*																												**-**	**-**
Mirounga	**Elephant seal**	*Phocidae*							**X**	**o**	**o**															**o**				**1**	**4**
Mus	**Mouse**	*Muridae*	**X**	**o**	**o**	**X**		**o**	**X**	**X**	**o**	**o**		**o**	**o**	**o**	**o**	**o**	**o**		**o**	**X**	**o**	**X**		**X**			**X**	**8**	**21**
Mustela	**Weasel**	*Mustelidae*		**X**	**X**	**X**		**X**	**X**	**o**	**o**		**X**	**X**	**o**											**X**				**8**	**11**
Myotis	**Bat**	*Vespertilionidae*							**X**					**o**					**o**	**o**				**o**		**X**	**X**			**3**	**7**
Myrmecophaga	**Giant anteater**	*Myrmecophagidae*										**X**	**o**																	**1**	**2**
Nasua	**Coati**	*Procyonidae*		**o**	**o**	**o**		**X**	**o**	**o**	**o**	**X**														**X**				**3**	**9**
Neophocaena	**Porpoise**	*Phocoenidae*									**o**																			**-**	**1**
Neotragus	**Antelope**	*Bovidae*	**o**	**o**	**o**	**o**	**o**	**o**	**o**	**o**	**o**								**o**			**o**	**o**	**o**	**o**	**o**				**-**	**15**
Nyctereutes	**Raccoon dog**	*Canidae*	**X**	**o**	**o**	**o**	**o**	**o**	**o**	**o**	**o**			**X**	**o**				**o**				**o**		**o**	**X**	**o**			**3**	**16**
Nycticebus	**Slow loris**	*Loridae*			**X**																									**1**	**1**
Odocoileus	**Deer**	*Cervidae*	**X**	**X**	**X**	**X**		**X**	**o**	**o**	**X**															**X**				**7**	**9**
Oreotragus	**Antelope**	*Bovidae*	**o**	**o**	**o**	**o**	**o**	**X**	**o**	**o**	**o**								**o**			**o**	**o**	**o**	**o**	**o**				**1**	**15**
Orycteropus	**Aardvark**	*Orycteropodidae*						**X**																		**X**				**2**	**2**
Oryctolagus	**European rabbit**	*Leporidae*	**o**	**o**		**X**			**X**	**o**	**o**	**X**											**o**	**o**		**X**				**4**	**10**
Oryx	**Oryx**	*Bovidae*	**o**	**o**	**X**	**o**	**o**	**X**	**o**	**o**	**X**								**o**			**o**	**X**	**o**	**o**	**o**				**4**	**15**
Otocolobus	**Pallas cat**	*Felidae*	**o**	**o**	**o**	**o**	**o**	**o**	**o**	**o**	**o**			**o**	**o**				**o**				**X**			**o**	**o**			**1**	**15**

Otocyon	**Bat-eared fox**	*Canidae*	o	o	o	o	o	o	o	o	o			o	o				o				o		o	X	o			1	16
Ovis	**Sheep**	*Bovidae*	X	o	o	o	X	X	o	X	X								X			o	X	X	o	X				9	15
Pachyuromys	**Gerbil**	*Muridae*	o	o	o	o		o	o	o	o	o		o	o	o	o	o	o		o	o	o	o		o			o	-	21
Pan	**Chimpanzee**	*Hominidae*	o	o	X	o	o	X	X	o	o	X	X	o	o	o	o	o	o	o	o	X	o	o	o	X	o	X	o	8	27
Panthera	**Great cats**	*Felidae*	X	o	X	o	X	X	o	o	o			o	X				X				o			o	o			6	15
Papio	**Baboon**	*Cercopithecidae*			X	o		X	X	o		o	X		o					X		X				o		X		7	12
Paradoxurus	**Palm civet**	*Viverridae*			o			X	o					o	o								o			o				1	7
Pedetes	**Spring hare**	*Pedetidae*																					X							1	1
Pelea	**Rhebok**	*Bovidae*	o	o	o	o	o	o	o	o	o								o			o	o	o	o	o				-	15
Peromyscus	**Mouse**	*Muridae*	X	X	o	X		o	o	X	o	o		o	o	X	o	o	o		o	o	o	o		o			o	5	21
Petaurillus	**Squirrel**	*Sciuridae*	o	o		o			o	o		o							o				o	o		o				-	10
Petaurista	**Flying squirrel**	*Sciuridae*	o	o		o			o	o		o							o				o	o		o				-	10
Petaurus	**Glider**	*Petauridae*							X																					1	1
Petrogale	**Wallaby**	*Macropodidae*	X					o		o																				1	3
Phacochoerus	**Warthog**	*Suidae*	X		X			X	o		o			o	o		o					o	X		o	X				5	12
Phascolarctos	**Koala**	*Phascolarctidae*																												-	-
Philander	**Opossum**	*Didelphidae*		o	o	o			X		o	o	o												o	o				1	9
Phodopus	**Rodent**	*Muridae*	o	o	o	o		o	o	o	o	o		o	o	o	o	o	o		o	o	o	o		o			o	-	21
Pongo	**Orangutan**	*Hominidae*	o	o	X	o	o	o	o	o	o	X	o	o	o	o	o	o	o	o	o	o	o	o	o	o	o	o	o	2	27
Potamochoerus	**African bush pig**	*Suidae*	X		X			X	o	o	o			o	o		o					o	o		o	o				3	13
Potos	**Kinkajou**	*Procyonidae*		o	o	o		o	o	o	o	o														o				-	9
Prionailurus	**Feline**	*Felidae*	o	o	o	o	o	X	o	o	o			X	o				o				o			o	o			2	15
Procapra	**Antelope**	*Bovidae*	o	o	o	o	o	o	o	o	o								o			o	o	o	o	o				-	15
Procavia	**Hyrax**	*Procaviidae*		o	X			o																		X				2	4

Procyon	**Raccoon**	*Procyonidae*		X	X	X		X	X	X	X	o														X				8	9
Psammomys	**Rodent**	*Muridae*	o	o	o	o		o	o	o	o	o		o	o	o	o	o	o		o	o	o	o		o			o	-	21
Pseudalopex	**Fox**	*Canidae*	X	o	o	o	o	o	o	o	o			o	o				o				o		o	X	o			2	16
Pseudocheirus	**Ringtailed possum**	*Pseudocheiridae*																												-	-
Pteronotus	**Bat**	*Mormoopidae*																								X				1	1
Pteronura	**Otter**	*Mustelidae*		o	o	o		o	o	o	o		o	o	o											o				-	11
Pteropus	**Bat**	*Pteropodidae*												o					o	o		o		o		X	X			2	7
Puma	**Feline**	*Felidae*	X	o	o	o	o	X	o	o	o			o	o				o				o			X	o			3	15
Pygathrix	**Langur**	*Cercopithecidae*			o	o		o	o	o		o	o		o					o		o				o		o		-	12
Raphicerus	**Antelope**	*Bovidae*	o	o	o	o	o	X	o	o	X								o			o	o	o	o	X				3	15
Rattus	**Rat**	*Muridae*	X	X	X	X		X	X	X	o	o		X	o	o	X	o	X		o	o	o	X		X			X	13	21
Ratufa	**Squirrel**	*Sciuridae*	X	o		o			o	o		o							o				o	o		o				1	10
Redunca	**Reedbuck**	*Bovidae*	o	o	X	o	o	X	o	o	o								o			o	X	o	o	X				4	15
Rhinolophus	**Bat**	*Rhinolophidae*												X			X		X	X				X		X	X			7	7
Rhipidomys	**Rodent**	*Muridae*	o	o	o	o		o	o	o	o	o		o	o	o	o	o	o		o	o	o	o		o			o	-	21
Rupicapra	**Chamois**	*Bovidae*	o	o	o	o	o	o	o	X	X								o			o	o	o	o	X				3	15
Saguinus	**Tamarin**	*Cebidae*			o	X						o	X													o			X	3	6
Saimiri	**Squirrel monkey**	*Cebidae*			X	X			X			X	X													o			o	5	7
Scapanus	**Mole**	*Talpidae*		X	o																					o				1	3
Sciurus	**Squirrel**	*Sciuridae*	o	X		X			X	X		o							X				o	o		X			X	7	11
Speothos	**Canine**	*Canidae*	o	o	o	o	o	o	o	o	o			o	o				o				o		o	o	o			-	16
Spermophilus	**Ground squirrel**	*Sciuridae*	o	X		X			o	X		o							o				o	o		X				4	10
Suricata	**Suricate**	*Herpestidae*			X																					X				2	2
Sylvicapra	**Bush duiker**	*Bovidae*	o	o	o	o	o	X	o	o	X								o			o	X	o	o	X				4	15

Syncerus	**Cape buffalo**	*Bovidae*	**X**	**o**	**X**	**o**	**o**	**X**	**o**	**o**	**X**								**X**			**o**	**X**	**o**	**o**	**o**				**6**	**15**
Tamandua	**Anteater**	*Myrmecophagidae*										**o**	**X**																	**1**	**2**
Tamiasciurus	**Squirrel**	*Sciuridae*	**o**	**X**		**X**			**o**	**o**		**o**							**o**				**o**	**o**		**o**				**2**	**10**
Taurotragus	**Eland**	*Bovidae*	**o**	**o**	**X**	**o**	**o**	**X**	**o**	**o**	**X**								**o**			**o**	**X**	**o**	**o**	**X**				**5**	**15**
Tolypeutes	**Armadillo**	*Dasypodidae*																								**o**				**-**	**1**
Trachypithecus	**Monkey**	*Cercopithecidae*			**o**	**o**		**o**	**o**	**o**		**o**	**o**		**o**					**o**		**o**				**o**		**o**		**-**	**12**
Tragelaphus	**Antelope**	*Bovidae*	**X**	**o**	**X**	**o**	**o**	**X**	**X**	**o**	**X**								**o**			**o**	**X**	**o**	**o**	**X**				**7**	**15**
Tragulus	**Mouse deer**	*Tragulidae*																												**-**	**-**
Trichosurus	**Brushtail possum**	*Phalangeridae*			**X**				**X**	**X**																				**3**	**3**
Tupaia	**Tree shrew**	*Tupaiidae*																												**-**	**-**
Tursiops	**Dolphin**	*Delphinidae*		**o**	**o**	**o**			**o**		**X**	**o**	**o**												**o**	**o**				**1**	**9**
Ursus	**Bear**	*Ursidae*		**X**		**X**		**X**	**X**	**X**	**X**															**X**				**7**	**7**
Viverra	**Civet**	*Viverridae*			**o**			**X**	**o**					**X**	**o**								**o**			**o**				**2**	**7**
Vulpes	**Fox**	*Canidae*	**X**	**X**	**X**	**o**	**o**	**o**	**X**	**X**	**X**			**X**	**o**				**o**				**X**		**o**	**X**	**o**			**9**	**16**
Wallabia	**Wallaby**	*Macropodidae*	**X**					**o**		**o**																				**1**	**3**
Xerus	**Squirrel**	*Sciuridae*	**o**	**X**		**o**			**o**	**o**		**o**							**o**				**X**	**o**		**X**				**3**	**10**
**Total affected genera†**			**41**	**24**	**48**	**31**	**5**	**57**	**35**	**20**	**32**	**13**	**16**	**8**	**4**	**1**	**2**	**-**	**13**	**4**	**1**	**5**	**27**	**6**	**4**	**78**	**5**	**6**	**7**		
**Total potentially affected genera‡**			**89**	**101**	**124**	**115**	**46**	**113**	**131**	**108**	**95**	**62**	**37**	**56**	**62**	**17**	**23**	**17**	**76**	**14**	**17**	**55**	**91**	**59**	**46**	**155**	**23**	**12**	**25**		
Legend: X = Risk Zoonosis identified in genus; o = Risk Zoonosis identified in different genus within same family.																															

*Rabies viruses includes the zoonotic lyssaviruses Australian bat lyssavirus, Duvenhage, European bat lyssavirus 1 and 2, Mokolo, and rabies (*234*); tick-borne encephalitis complex includes Kyasanur Forest disease, Omsk hemorrhagic fever, and tickborne encephalitis (*234*); Ebolaviruses include Bundibugyo, Côte d'Ivoire, Reston, Sudan and Zaire (*234*); epidemiologically relevant South American hemorrhagic fever arenaviruses include Guanarito, Junin, Machupo, and Sabia (*234*); hantaviruses associated with HFRS include Dobrava, Hantaan, Puumala, Saaremaa, and Seoul (*234*); hantaviruses associated with HCPS include Andes, Bayou, Black Creek Canal, Laguna Negra, New York, and Sin Nombre (*234*); *Mycobacterium tuberculosis* complex species are *M. africanum, M. bovis, M. bovis BCG, M. caprae, M. microti, M. pinnipedii,* and *M. tuberculosis hominis* (*235*).
†Risk zoonosis identified in genus.
‡Risk zoonosis identified in different genus within same family.

References

1. United States Animal Health Association. “The Grey Book” – Foreign Animal Diseases. [Cited 2007 Apr 26]. Available from http://www.usaha.org/pubs/fad.pdf

2. PathInfo. Virginia Bioinformatics Institute - PathPort: The Pathogen Portal Project. [Cited 2007 Apr 26]. Available from http://pathport.vbi.vt.edu/pathinfo/index.php

3. Centers for Disease Control and Prevention. Biosafety in microbiological and biomedical laboratories (BMBL), 4th ed [cited 2007 Apr 5]. Available from http://www.cdc.gov/OD/ohs/biosfty/bmbl4/bmbl4toc.htm

4. Ergönül O. Crimean-Congo haemorrhagic fever. Lancet Infect Dis 2006; 6(4): 203-214.

5. Shepherd AJ, Swanepoel R, Shepherd SP, McGillivray GM, Searle LA. Antibody to Crimean-Congo hemorrhagic fever virus in wild mammals from southern Africa. Am J Trop Med Hyg 1987; 36(1) 133-142.

6. Whitehouse CA. Crimean-Congo hemorrhagic fever. Antivir Res 2004; 63(3): 145-160.

7. World Organization for Animal Health. Animal Diseases Data [cited 2007 Apr 26]. Available from http://www.oie.int/eng/maladies/en_alpha.htm?e1d7

8. Anderson EC, Rowe LW. The prevalence of antibody to the viruses of bovine virus diarrhoea, bovine herpes virus 1, rift valley fever, ephemeral fever and bluetongue and to *Leptospira* sp. in free-ranging wildlife in Zimbabwe. Epidemiol Infect 1998; 121: 441-449.

9. Boiro I, Konstaninov OK, Numerov AD. Isolation of Rift Valley fever virus from bats in the Republic of Guinea. Bull Soc Pathol Exot Filiales 1987; 80(1): 62-67.

10. Davies FG, Martin V. Recognizing Rift Valley fever. FAO Animal Health Manual No. 17. 2003 [cited 2007 Apr 8]. Available from http://www.fao.org/DOCREP/006/Y4611E/y4611e00.htm

11. Youssef BZ, Donia HA. The potential role of *Rattus rattus* in enzootic cycle of Rift Valley Fever in Egypt. 1-Detection of RVF antibodies in *R. rattus* blood samples by both enzyme linked immuno sorbent assay (ELISA) and immuno-diffusion technique (ID). J Egypt Public Health Assoc 2001; 76(5-6): 431-441.

12. Kansas State University – National Agricultural Security Center. Rift Valley fever fact sheet [cited 2007 Apr 8]. Available from http://nabc.ksu.edu/content/factsheets/category/Rift%20Valley%20Fever

13. House C, Alexander KA, Kat PW, O'Brien SJ, Mangiafico J. Serum antibody to Rift Valley fever virus in African carnivores. Ann NY Acad Sci 1996; 791(1): 345–349.

14. United Kingdom Department of Environment, Food and Rural Affairs (DEFRA). International Animal Health Division. Preliminary outbreak assessment: Rift Valley Fever in Kenya 1/11/07 [cited 2007 Apr 5]. Available from http://www.defra.gov.uk/animalh/diseases/monitoring/pdf/rvf-kenya.pdf

15. Public Health Agency of Canada – Office of Laboratory Security. Material safety data sheet: Infectious substances [cited 2007 Apr 8]. Available from http://www.phac-aspc.gc.ca/msds-ftss/msds89e.html

16. Fulhorst CF, Ksiazek TG, Peters CJ, Tesh RB. Experimental infection of the cane mouse *Zygodontomys brevicauda* (Family Muridae) with Guanarito virus (Arenaviridae), the etiologic agent of Venezuelan hemorrhagic fever. J Infect Dis 1999; 180: 966–969.

17. Centers for Disease Control and Prevention. Tick-borne encephalitis [cited 2007 Apr 6]. Available from http://www.cdc.gov/ncidod/dvrd/spb/mnpages/dispages/TBE.htm

18. Bakhvalova VN, Dobrotvorsky AK, Panov VV, Matveeva VA, Tkachev SE, Morozova OV. Natural tick-borne encephalitis virus infection among wild small mammals in the southeastern part of western Siberia, Russia. Vector-Borne Zoonot 2006; 6(1): 32 -41.

19. Barrett ADT, Higgs S. Yellow fever: A disease that has yet to be conquered. Annu Rev Entomol 2007; 52: 209–229.

20. Salas RA, Garcia CZ, Liria J, Barrera R, Navarro JC, Medina G, et al. Ecological studies of enzootic Venezuelan equine encephalitis in north-central Venezuela, 1997–1998. Am J Trop Med Hyg 2001; 64(1, 2): 84–92.

21. Ubico SR, McLean RG. Serologic survey of neotropical bats in Guatemala for virus antibodies. J Wildl Dis 1995; 31(1): 1-9.

22. Calisher CH, Maness KS. Laboratory studies of Venezuelan equine encephalitis virus in equines, Texas, 1971. J Clin Microbiol 1975; 2(3): 198-205.

23. Rouquet P, Froment JM, Bermejo M, Kilbourn A, Karesh W, Reed P, et al. Wild animal mortality monitoring and human Ebola outbreaks, Gabon and Republic of Congo, 2001– 2003. Emerg Infect Dis 2005; 11(2): 283-290.

24. Leroy EM, Kumulungui B, Pourrut X, Rouquet P, Hassanin A, Yaba P, et al. Fruit bats as reservoirs of Ebola virus. Nature 2005; 435(7068): 575-6.

25. Georges-Courbot MC, Sanchez A, Lu CY, Baize S, Leroy E, Lansout-Soukate J, et al. Isolation and phylogenetic characterization of Ebola viruses causing different outbreaks in Gabon. Emerg Infect Dis 1997; 3(1): 59-62.

26. Peters CJ, LeDuc JW. An introduction to Ebola: The virus and the disease. J Infect Dis 1999; 179(Suppl 1): ix-xvi.

27. Iowa State University - Institute for International Cooperation in Animal Biologics. Viral Hemorrhagic Fevers–Ebola and Marburg [cited 2007 Apr 6]. Available from http://www.cfsph.iastate.edu/Factsheets/pdfs/viral_hemorrhagic_fever_filovirus.pdf

28. Stephensen CB, Jacob JR, Montali RJ, Holmes KV, Muchmore E, Compans RW, et al. Isolation of an arenavirus from a marmoset with callitrichid hepatitis and its serologic association with disease. J Virol 1991; 65(8): 3995–4000.

29. Amman BR, Pavlin BI, Albariño CG, Comer JA, Erickson BR, Oliver JB, et al. Pet rodents and fatal lymphocytic choriomeningitis in transplant patients. Emerg Infect Dis 2007; 13(5): 719-725.

30. Li W, Shi Z, Yu M, Ren W, Smith C, Epstein JH, et al. Bats are natural reservoirs of SARS-like coronaviruses. Science 2005; 310(5748): 676-9.

31. Steppan SJ, Hamm SM. Xerini: African ground squirrels. The Tree of Life Web Project [cited 2007 Apr 20]. Available from http://tolweb.org/Xerini/16817/2006.08.10

32. Marker LL. Aspects of cheetah (Acinonyx jubatus) biology, ecology and conservation strategies on Namibian farmlands. Doctoral thesis, Lady Margaret Hall, University of Oxford, 2002 [cited 2007 Apr 20]. Available from http://www.carnivoreconservation.org/files/thesis/marker_2002_ phd.pdf

33. Wempe JM. Rabies in a pronghorn antelope, *Antilocapra americana*. J Wildl Dis 1976; 12: 347-348.

34. Riley PY, Chomel BB. Hedgehog zoonoses. Emerg Infect Dis 2005; 11(1): 1-5.

35. Favoretto SR, de Mattos CC, Morais NB, Alves Araújo FA, de Mattos CA. Rabies in marmosets (*Callithrix jacchus*), Ceará, Brazil. Emerg Infect Dis 2001; 7(6): 1062-1065.

36. Ontario Ministry of Natural Resources. Manual of common parasites, diseases and anomalies of wildlife in Ontario. Ontario: 1998, Queens Printer [cited 2007 Apr 28]. Available from http://www.unbc.ca/nlui/wildlife_diseases/booklet.htm

37. Aghomo HO, Ako-Nai AK, Oduye OO, Tomori O, Rupprecht CE. Detection of rabies virus antibodies in fruit bats (*Eidolon helvum*) from Nigeria. J Wildl Dis 1990; 26(2): 258-261.

38. Greenhall AM. 1961. Bats in Agriculture. Ministry of Agriculture, Trinidad and Tobago. From: “Rabies” in Wikipedia, www.wikipedia.org

39. Eidson M, Matthews SD, Willsey AL, Cherry B, Rudd RJ, Trimarchi CV. Rabies virus infection in a pet guinea pig and seven pet rabbits. J Am Vet Med Assoc 2005; 227(6): 932-935.

40. Krebs JW, Williams SM, Smith JS, Rupprecht CE, Childs JE. Rabies among infrequently reported mammalian carnivores in the United States, 1960-2000. J Wildl Dis 2003; 39(2): 253-261.

41. Johnson N, Black C, Smith J, Un H, McElhinney LM, Aylan O, Fooks AR. Rabies emergence among foxes in Turkey. J Wildl Dis 2003; 39(2): 262-270.

42. Krebs JW, Mondul AM, Rupprecht CE, Childs JE. Rabies surveillance in the United States during 2000. J Am Vet Med Assoc 2001; 219(12): 1687-1699.

43. Berry HH. Surveillance and control of anthrax and rabies in wild herbivores and carnivores in Namibia. Rev Sci Tech 1993; 12(1): 137-146.

44. Svrcek S, Ondrejka R, Mlynarcikova K, Svec J. Rabies in the common hamster (*Cricetus cricetus*) in Slovakia. Vet Med (Praha) 1984; 29(11): 643-652.

45. Leffingwell LM, Neill SU. Naturally acquired rabies in an armadillo (*Dasypus novemcinctus*) in Texas. J Clin Microbiol 9189; 27: 174-175.

46. Wilamaratne O, Kodikara DS. First reported case of elephant rabies in Sri Lanka. Vet Rec 1999; 144(4): 98.

47. Arizona Department of Health Services – Office of Infectious Disease Services. Vector borne and zoonotic disease newsletter – Winter 2001 [cited 2007 Apr 5]. Available from http://www.azdhs.gov/phs/oids/vector/zoonotic1.htm

48. World Organisation for Animal Health. Epidemiological review of wildlife diseases. World Animal Health – 2004 [cited 2007 Apr 29]. Available from ftp://ftp.oie.int/SAM/2004/FAUNE_A.pdf

49. Botros BA, Moch RW, Kerkor M, Helmy I. Rabies in the Arab Republic of Egypt: III. Enzootic rabies in wildlife. J Trop Med Hyg 1977; 80(3): 59-62.

50. Sheeler-Gordon LL, Smith JS. Survey of bat populations from Mexico and Paraguay for rabies. J Wildl Dis 2001; 37(3): 582-593.

51. Davidson ML. Rabies in a llama. Vet Med 1998; 10: 934-936.

52. Mikota S. Elephant Care International fact sheet: Rabies [cited 2007 Apr 20]. Available from http://www.elephantcare.org/rabies.htm

53. FORTH – For Travelers' Health. Rabies [cited 2007 Apr 20]. Available from http://www.forth.go.jp/mhlw/animal/down/1dog/information-update.pdf

54. Bishop GC, Durrheim DN, Kloeck PE, Godlonton JD, Bingham J, Speare R. Rabies: Guide for the medical, veterinary and allied health professions. Republic of South Africa Rabies Advisory Group [cited 2007 Apr 20]. Available from http://www.nda.agric.za/docs/rabiesB5.pdf

55. World Health Organization Technical Report Series #931. WHO expert consultation on rabies [cited 2007 Apr 20]. Available from http://www.who.int/rabies/trs931_%2006_05.pdf

56. Schoop U. *Praomys* (*Mastomys*) *natalensis*: an African mouse capable of sustaining persistent asymptomatic rabies infection. Ann Microbiol (Paris) 1997; 128(2): 289-298.

57. Jadav KK. Rabies in captive sloth bears (*Melursus ursinus*). 1st Scientific Meeting of the Asian Zoo & Wildlife Medicine 2005 [cited 2007 Apr 27]. Available from http://blog.goo.ne.jp/jszwd

58. Nel LH, Sabeta CT, von Teichman B, Jaftha JB, Rupprecht CE, Bingham J. Mongoose rabies in southern Africa: A re-evaluation based on molecular epidemiology. Virus Res 2005; 109(2): 165-173.

59. Favoretto SR, Carrieri ML, Cunha EMS, Aguiar EAC, Silva LH, Sodre MM, et al. Antigenic typing of Brazilian rabies virus samples isolated from animals and humans, 1989-2000. Rev Inst Med Trop S Paulo 2002; 44(2): 91-95.

60. Krebs JW, Noll HR, Rupprecht CE, Childs JE. Rabies surveillance in the United States during 2001. J Am Vet Med Assoc 2002; 221(12): 1690-1701.

61. Potter TM, Hanna JA, Freer L. Human North American river otter (*Lontra canadensis*) attack. Wild Environ Med 2007; 18(1): 41-44.

62. Shulaw W, James RE. Ohio State University Extension fact sheet: Rabies prevention in livestock [cited 2007 Apr 27]. Available from http://ohioline.osu.edu/vme-fact/0001.html

63. Miot MR, Sikes RK, Silberman MS. Rabies in a chimpanzee. J Am Vet Med Assoc 1973; 162(1): 54.

64. Centers for Disease Control and Prevention. Epidemiologic notes and reports rabies in a javelina – Arizona. MMWR 1986; 35(35): 555-6,561.

65. Merck Veterinary Manual – Viral Diseases. Marine mammals: Viral diseases [cited 2007 Apr 27]. Available from http://www.merckvetmanual.com/mvm/index.jsp?cfile=htm/bc/170811.htm

66. Cunha EMS, da Silva LHQ, Lara MCCSH, Nassar AFC, Albas A, Sodre MM, et al. Bat rabies in the north-northwestern regions of the state of Sao Paulo, Brazil: 1997-2002. Rev Saude Publica 2006; 40(6): 1082-1086.

67. Brandao N. Phylogeny of a rabies virus variant with an unusual antigenic pattern isolated from platyrrhinus sp from northern Brazil. The XV International Conference Rabies in the Americas (RITA XV), 2004 [cited 2007 Apr 28]. Available from http://rita15.rabies-in-the-americas.org/I/program.htm

68. Sato G, Kobayashi Y, Shoji Y, Sato T, Itou T, Ito FH, et al. Molecular epidemiology of rabies from Maranhao and surrounding states in the northeastern region of Brazil. Arch Virol 2006; 151(11): 2243-2251.

69. Pal SR, Arora B, Chhuttani PN, Broor S, Choudhury S, Joshi RM, et al. Rabies virus infection of a flying fox bat, *Pteropus policephalus* in Chandigarh, Northern India. Trop Geogr Med 1980; 32(3): 265-7.

70. Arkanimals.com. Cougar incidents & mountain lion attacks expert comments. Available at: www.arkanimals.com/dlg/cougar.htm

71. Centers for Disease Control and Prevention. Cases of rabies in the United States and Puerto Rico, by state and category, 2001 [cited 2007 Apr 28]. Available from http://www.cdc.gov/ncidod/dvrd/rabies/Professional/publications/Surveillance/Surveillance01/Table1-01.htm

72. Padilla M, Dowler RC. *Tapirus terrestris*. Mammalian Species, No. 481. American Society of Mammalogists 1994 [cited 2007 Apr 5]. Available from http://links.jstor.org/sici?sici=0076-3519(19941202)5:481%3C1:TT%3E2.0.CO;2-G

73. Mutinelli F, Lattuada E, Mustoni A. Detection of rabies antibodies in a brown bear (*Ursus arctos*). Vet Rec 2001; 149(25): 779-780.

74. University of Connecticut Health Center – Animal Care Committee. Cercopithecine herpesvirus 1 (Herpes B virus) [cited 2007 Apr 5]. Available from http://clacc.uchc.edu/ACC/Occupational%20Health%20and%20Safety/HerpesB.htm

75. Acha PN, Szyfres B. Kyasanur Forest disease. In: Zoonoses and communicable diseases common to man and animals, third edition - Volume II: Chlamydioses, rickettsioses, and viroses [cited 2007 Apr 6]. Available from http://athena.bioc.uvic.ca/bioDoc/flaviviridae/flavivirus/kyasanur.pdf

76. Centers for Disease Control and Prevention. Hendra virus disease and Nipah virus encephalitis [cited 2007 Apr 8]. Available from http://www.cdc.gov/ncidod/dvrd/spb/mnpages/dispages/nipah.htm

77. Herbreteau V, Gonzalez JP, Hugot JP. Implication of phylogenetic systematics of rodent-borne hantaviruses allows understanding of their distribution. Ann NY Acad Sci 2006; 1081: 39-56.

78. Kariwa H, Lokugamage K, Lokugamage N, Miyamoto H, Yoshii K, Nakauchi M, et al. A comparative epidemiological study of hantavirus infection in Japan and Far East Russia. Jpn J Vet Res 2007; 54(4): 145-161.

79. Focosi D. Molecular Medicine [cited 2007 Apr 10]. Available from http://focosi.altervista.org

80. Centers for Disease Control and Prevention. Human monkeypox – Kasai Oriental, Zaire, 1996-1997. MMWR 1997; 46(14): 304-307.

81. Khodakevich L, Szczeniowski M, Manbu-ma-Disu, Jezek Z, Marennikova S, Nakano J, et al. The role of squirrels in monkeypox virus transmission. Trop Geogr Med 1987; 39(2): 115-122.

82. Cyranoski D. Bird flu data languish in Chinese journals. Nature 2004; 430(7003): 955.

83. Clegg SB, Turnbul PCB, Foggin CM, Lindeque PM. Massive outbreak of anthrax in wildlife in the Malilangwe Wildlife Reserve, Zimbabwe. Vet Rec 2007; 160: 113-118.

84. Nishi JS, Ellsworth TR, Lee N, Dewar D, Elkin BT, Dragon DC. Northwest Territories. An outbreak of anthrax (*Bacillus anthracis*) in free-roaming bison in the Northwest Territories, June-July 2006. Can Vet J 2007; 48(1): 37-38.

85. Ikede BO, Falade S, Golding RR. Anthrax in captive carnivores in Ibadan, Nigeria. J Wildl Dis 1976; 12(2): 130-132.

86. Louisiana State University – World Health Organization Collaborating Center for Remote Sensing and Geographic Information Systems for Public Health. World Anthrax Data Site [cited 2007 Apr 4]. Available from http://www.vetmed.lsu.edu/whocc/AnthraxStats2001-DataFiles/Asia/India.htm

87. Shiferaw F, Abditcho S, Gopilo A, Laurenson MK. Anthrax outbreak in Mago National Park, southern Ethiopia. Vet Rec 2002; 150: 318-320.

88. Hugh-Jones ME, de Vos V. Anthrax and wildlife. Rev Sci Tech Off Int Epiz 2002; 21(2): 359-383.

89. Bookrags.com. Anthrax summary pack [cited 2007 Apr 28]. Available from http://www.bookrags.com/Anthrax

90. Brashares JS, Arcese P, Sam MK, Coppolillo PB, Sinclair ARE, Balmford A. Bushmeat hunting, wildlife declines, and fish supply in West Africa. Science 2004; 306(5699): 1180-1183.

91. Pura Petspourri. Outbreaks of anthrax [cited 2007 Apr 28]. Available from http://www.petspourri.com/trends05.htm

92. Dodgson SJ, Forster RE II. Carbonic anhydrase activity of intact erythrocytes from seven mammals. J Appl Physiol 1983; 55(4): 1292-1298.

93. U.S. Department of the Interior – Minerals Management Service. Population dynamics and biology of the California sea otter (*Enhydra lutris nereis*) at the southern end of its range. 2006 [cited 2007 Apr 28]. Available from http://www.coastalresearchcenter.ucsb.edu/cmi/files/2006-007.pdf

94. McDonald WL, Jamaludin R, Mackereth G, Hansen M, Humphrey S, Short P, et al. Characterization of a *Brucella* sp. strain as a marine-mammal type despite isolation from a patient with spinal osteomyelitis in New Zealand. J Clin Microbiol 2006; 44(12): 4363-4370.

95. Nielsen K, Duncan JR. Animal Brucellosis. 1990. CRC Press [cited 2007 Apr 30]. Available from http://books.google.com/books?id=QQFDHBpNnTQC&pg=RA1-A143&lpg=RA1-PA143&dq=phacochoerus+brucella&sig=lmsalk7okERH4OAz9a2K16vZOuo#PPA1,M1

96. Lundervold M. Infectious diseases of saiga antelopes and domestic livestock in Kazakhstan. Doctoral thesis, University of Warwick, UK, 2001 [cited 2007 Apr 2]. Available from http://www.iccs.org.uk/papers/lundervold-thesis.pdf

97. Higgins R. Bacteria and fungi of marine mammals: A review. Can Vet J 2000; 41: 105-116.

98. Schiemann B, Staak C. *Brucella melitensis* in impala (*Aepyceros melampus*). Vet Rec 1971; 88(13): 344.

99. Ito FH, Vasconcellos SA, Bernardi F, Nascimento AA, Labruna MB, Arantes IG. Serological evidence of brucellosis, leptospirosis and parasitism by ixodid ticks in wild animals of the pantanal of Mato Grosso do Sul, Brazil. Ars-Veterinaria 1998; 14(3): 302-310.

100. Levy CE, Gage KL. Plague in the United States, 1995-1997. Infect Med 1999; 16(1): 54-64.

101. Ruiz A. Plague in the Americas. Emerg Infect Dis 2001; 7(3 Suppl.): 539-540.

102. Salkeld DJ, Stapp P. Seroprevalence rates and transmission of plague (*Yersinia pestis*) in mammalian carnivores. Vector-Borne Zoonot 2006; 6(3): 231-239.

103. Nelson BC. Plague studies in California – the roles of various species of sylvatic rodents in plague ecology in California. Proceedings of the 9th Vertebrate Pest Conference, 1980 [cited 2007 Apr 6]. Available from http://digitalcommons.unl.edu/vpc9/30

104. Kilonzo B, Mhina J, Sabuni C, Mgode G. The role of rodents and small carnivores in plague endemicity in Tanzania. Belg J Zool 2005; 135(Suppl): 119-125.

105. Woldehiwet Z. Q fever (coxiellosis): epidemiology and pathogenesis. Res Vet Sci 2004; 77(2): 93-100.

106. Marrie TJ, Schlech WF III, Williams JC, Yates L. Q fever pneumonia associated with exposure to wild rabbits. Lancet 1986; 1(8478): 427-429.

107. Pope JH, Scott W, Dwyer R. *Coxiella burnetii* in kangaroos and kangaroo ticks in western Queensland. Aust J Exp Biol Med Sci 1960; 38: 17-27.

108. Riemann HP, Behymer DE, Franti CE, Crabb C, Schwab RG. Survey of Q-fever agglutinins in birds and small rodents in Northern California, 1975-76. J Wildl Dis 1979; 15(4): 515-523.

109. McQuiston JH, Childs JE. Q fever in humans and animals in the United States. Vector-Borne Zoonot 2002; 2(3): 179-191.

110. American Veterinary Medical Association. Q fever backgrounder. 2006 [cited 2007 Apr 3]. Available from http://www.avma.org/public_health/biosecurity/qfever_bgnd.asp

111. Secretariat of the Pacific Community. Q fever [cited 2007 Apr 3]. Available from http://www.spc.int/rahs/Manual/Multiple_Species/QFEVERE.HTM

112. Webster JP, Lloyd G, Macdonald DW. Q fever (*Coxiella burnetii*) reservoir in wild brown rat (Rattus norvegicus) populations in the UK. Parasitology 1995; 110(Pt 1): 31-35.

113. Enright JB, Franti CE, Behymer DE, Longhurst WM, Dutson VJ, Wright ME. Coxiella burneti in a wildlife-livestock environment: Distribution of Q fever in wild animals. Am J Epidemiol 1971; 94(1): 79-90.

114. Ejercito CL, Cai L, Htwe KK, Taki M, Inoshima Y, Kondo T, et al. Serological evidence of *Coxiella burnetii* infection in wild animals in Japan. J Wildl Dis 1993; 29(3): 481-484.

115. Gallagher J, Macadam I, Sayer J, van Lavieren LP. Pulmonary tuberculosis in free-living lechwe antelope in Zambia. Trop Anim Health Pro 1972; 4(4): 204-213.

116. Allgood MA, Price GT. Isoniazid therapy of tuberculosis in baboons. Primates 1971; 12(1): 81-90.

117. Michel AL, Bengis RG, Keet DF, Hofmeyer M, Klerk LM, Cross PC, et al. Wildlife tuberculosis in South African conservation areas: implications and challenges. Vet Microbiol 2006; 112(2-4): 91-100.

118. Serraino A, Marchetti G, Sanguinetti V, Rossi MC, Zanoni RG, Catozzi L, et al. Monitoring of transmission of tuberculosis between wild boars and cattle: genotypical analysis of strains by molecular epidemiology techniques. J Clin Microbiol 1999; 37(9): 2766-2771.

119. Cleaveland S, Mlengeya T, Kazwala RR, Michel A, Kaare MT, Jones SL, et al. Tuberculosis in Tanzanian wildlife. J Wildl Dis 2005; 41(2): 446-453.

120. Payeur JB, Church S, Mosher L, Robinson-Dunn B, Schmitt S, Whipple D. Bovine tuberculosis in Michigan wildlife. Ann NY Acad Sci 2002; 969: 259-261.

121. Pinto MR, Jainudeen MR, Panabokke RG. Tuberculosis in a domesticated Asiatic elephant *Elephas maximus*. Vet Rec 1973; 93(26): 662-664.

122. Gorton RJ. A Study of tuberculosis in hedgehogs so as to predict the location of tuberculous possums. Masters thesis, Massey University, NZ, 1998 [cited 2007 Apr 20]. Available from http://epicentre.massey.ac.nz/Portals/0/EpiCentre/Downloads/Publications/Thesis/RobynGortonMVS.pdf

123. Martin-Atance P, Palomares F, Gonzalez-Candela M, Revilla E, Cubero MJ, Calzada J, et al. Bovine tuberculosis in a free ranging red fox (*Vulpes vulpes*) from Doñana National Park (Spain). J Wildl Dis 2005; 41(2): 435-436.

124. Mangold BJ, Cook RA, Cranfield MR, Huygen K, Godfrey HP. Detection of elevated levels of circulating antigen 85 by dot immunobinding assay in captive wild animals with tuberculosis. J Zoo Wildl Med 1999; 30(4): 477-483.

125. Wilson P, Weavers E, West B, Taylor M, Kavanagh J, Jones P. *Mycobacterium bovis* infection in primates in Dublin Zoo: epidemiological aspects and implications for management. Lab Anim 1984; 18(4): 383-387.

126. Lewerin SS, Olsson SL, Eld K, Roken B, Ghebremichael S, Koivula T, et al. Outbreak of *Mycobacterium tuberculosis* infection among captive Asian elephants in a Swedish zoo. Vet Rec 2005; 156(6): 171-175.

127. University of Wisconsin-Madison – National Primate Research Center. Tuberculosis [cited 2007 Apr 20]. Available from http://pin.primate.wisc.edu/aboutp/pets/tb.html

128. Leathers CW, Hamm TE Jr. Naturally occurring tuberculosis in a squirrel monkey and a cebus monkey. J Am Vet Med Assoc 1976; 169(9): 909-911.

129. Michel AL. Implications of tuberculosis in African wildlife and livestock. Ann NY Acad Sci 2002; 969: 251-255.

130. Cooke MM, Jackson R, Coleman JD. Tuberculosis in a free-living brown hare (*Lepus europaeus occidentalis*). New Zeal Vet J 1993; 41(3): 144-146.

131. Delahay RJ, Cheeseman CL, Clifton-Hadley RS. Wildlife disease reservoirs: the epidemiology of *Mycobacterium bovis* infection in the European badger (*Meles meles*) and other British mammals. Tuberculosis (Edinb) 2001; 81(1-2): 43-49.

132. Michalak K, Austin C, Diesel S, Bacon JM, Zimmerman P, Maslow JN. *Mycobacterium tuberculosis* infection as a zoonotic disease: transmission between humans and elephants. Emerg Infect Dis 1998; 4(2): 283-287.

133. Michel AL. *Mycobacterium tuberculosis*: an emerging disease of free-ranging wildlife. Emerg Infect Dis 2002; 8(6): 598-601.

134. Corner LAL. The role of wild animal populations in the epidemiology of tuberculosis in domestic animals: how to assess the risk. Vet Microbiol 2006; 112: 303-312.

135. Bunnell JE, Hice CL, Watts DM, Montrueil V, Tesh RB, Vinetz JM. Detection of pathogenic *Leptospira* spp. infections among mammals captured in the Peruvian Amazon Basin region. Am J Trop Med Hyg 2000; 63(5,6): 255-258.

136. Richardson DJ, Gauthier JL. A serosurvey of leptospirosis in Connecticut peridomestic wildlife. Vector-Borne Zoonot 2003; 3(4) 187-193.

137. Anderson EC, Rowe LW. The prevalence of antibody to the viruses of bovine virus diarrhoea, bovine herpes virus 1, rift valley fever, ephemeral fever and bluetongue and to Leptospira sp in free-ranging wildlife in Zimbabwe. Epidemiol Infect 1998; 121: 441-449.

138. Fennestad KL, Borg-Petersen C. Leptospirosis in Danish wild animals. J Wildl Dis 1972; 8: 343-351.

139. Diesch SL, McCulloch WF, Braun JL, Davis JR. Detection and ecology of leptospirosis in Iowa wildlife. J Wildl Dis 1970; 6: 275-288.

140. McKiel JA, Cousineau JG, Hall RR. Leptospirosis in wild animals in eastern Canada with particular attention to the disease in rats. Can J Comp Med Vet Sci 1961; 25: 15-18.

141. Sebek Z, Sixl W, Reinthaler F, Abdel-Nabi O, Stunzner D, Schneeweiss W, et al. Leptospirosis in the Melut district-upper Nile province (south Sudan)-an overview. Geogr Med Suppl 1989; 5: 161-178.

142. Colegrove KM, Lowenstine LJ, Gulland FMD. Leptospirosis in northern elephant seals (*Mirounga angustirostris*) stranded along the California coast. J Wildl Dis 2005; 41(2): 426-430.

143. Stoyanova N, Tokarevich N, Gracheva L, Volkova G, Gracheva N, Kravchenko S, et al. Leptospirosis in north-west Russia. EpiNorth. 2004 [cited 2007 Apr 21]. Available from http://www.epinorth.org/eway/default0.asp?Pid=230&oid=0&e=0&trg=MainArea_5260&MainArea_5260=5273:44619::1:5262:1:5260;::10:0:0

144. Santa Rosa CA, Sulzer CR, Giorgi W, da Silva AS, Yanaquita RM, Lobao AO. Leptospirosis in wildlife in Brazil: isolation of a new serotype in the pyrogenes group. Am J Vet Res 1975; 36(9): 1363-1365.

145. Mendoza P, Mayor P, Galvez HA, Cespedes MJ, Jori F. Antibodies against *Leptospira* spp. In captive collared peccaries, Peru. Emerg Infect Dis 2007; 13(5): 793-794.

146. de Lisle GW, Almand KB, Julian AF, Wallace J. Leptospirosis in the opossum (*Trichosurus vulpecula*). New Zeal Vet J 1975; 23(9): 215-216.

147. Anderson DC, Geistfeld JG, Maetz HM, Patton CM, Kaufmann AF. Leptospirosis in zoo workers associated with bears. Am J Trop Med Hyg 1978; 27(1 Pt 1): 210-211.

148. CBWInfo. Factsheets on Chemical and Biological Warfare Agents: Glanders [cited 2007 Apr 22]. Available from http://www.cbwinfo.com/Biological/Pathogens/BMa.html

149. Berrada ZL, Goethert HK, Telford SR III. Raccoons and skunks as sentinels for enzootic tularemia. Emerg Infect Dis 2006; 12(6): 1019-1021.

150. Zhang F, Liu W, Chu MC, He J, Duan Q, Wu XM, et al. *Francisella tularensis* in rodents, China. Emerg Infect Dis 2006; 12(6): 994-996.

151. Morner T, Sandstrom G, Mattsson R, Nilsson PE. Infections with *Francisella tularensis* biovar palaearctica in hares (*Lepus tumidus*, *Lepus europaeus*) from Sweden. J Wildl Dis 1988; 24(3): 422-433.

152. Audet AM, Robbins CB, Lariviere S. *Alopex lagopus*. Mammalian Species 2002; 713: 1-10.

153. Posthaus H, Welle M, Morner T, Nicolet J, Kuhnert P. Tularemia in a common marmoset (*Callithrix jacchus*) diagnosed by 16S rRNA sequencing. Vet Microbiol 1998; 61(1): 145-150.

154. Emmons RW, Ruskin J, Bissett ML, Uyeda DA, Wood RM, Lear CL. Tularemia in a mule deer. J Wildl Dis 1976; 12(3): 459-463.

155. University of California Santa Barbara - Institutional Animal Care and Use Committee. Zoonotic diseases [cited 2007 Apr 22]. Available from http://research.ucsb.edu/connect/acc/policy.html

156. Feldhamer GA, Whittaker JC, Bloemer SR. 2002. Often unseen fauna: small mammals and mesocarnivores on Land between the Lakes. Pp. 421-435 in J. S. Fralish and E. W. Chester, (eds.). Land Between The Lakes, Kentucky and Tennessee: Four Decades of Tennessee Valley Authority Stewardship. Austin Peay State University Center for Field Biology, Clarksville, TN [cited 2007 Apr 22]. Available from http://campus.pc.edu/~jwhittak/Feldhameretal2002.pdf

157. U. S. Department of Agriculture – Agricultural Research Service. Information resources on the North American opossum (*Didelphis virginiana*): A Bibliography on its natural history and use in biomedical research. Compiled by: Krause WJ [cited 2007 Apr 22]. Available from http://www.nal.usda.gov/awic/pubs/opossum.htm

158. Wobeser G, Ngeleka M, Appleyard G, Bryden L, Mulvey MR. Tularemia in deer mice (*Peromyscus maniculatus*) during a population irruption in Saskatchewan, Canada. J Wildl Dis 2007; 43(1): 23-31.

159. Koprowski JL. Pine squirrel (*Tamiasciurus hudsonicus*): A Technical conservation assessment. Prepared for the USDA Forest Service, Rocky Mountain Region, Species Conservation Project, 2005 [cited 2007 Apr 22]. Available from http://www.fs.fed.us/r2/projects/scp/assessments/pinesquirrel.pdf

160. Beckwith CS. Tularemia as a cause of fever in a squirrel monkey. J Am Vet Med Assoc 2006; 229(2): 269-273.

161. Farhang-Azad A, Mescerjakova I, Neronov V. Afghan hedgehog, a new reservoir of tularemia. Bull Soc Pathol Exot Filiales 1973; 66(2): 266-269.

162. Jenkins DJ, Macpherson CNL. Transmission ecology of echinococcus in wild-life in Australia and Africa. Parasitology 2003; 127: S63-S72.

163. Rodrigues-Silva R, Peixoto JRV, de Oliveira RMF, MagalhaesPinto R, Gomes DC. An Autochthonous case of *Echinococcus vogeli* Rausch & Bernstein, 1972 polycystic echinococcosis in the state of Rondônia, Brazil. Mem I Oswaldo Cruz 2002; 97(1): 123-126.

164. Courtenay O, Maffei L. Crab-eating fox *Cerdocyon thous* (Linnaeus, 1766) [cited 2007 Apr 28]. Available from http://www.canids.org/species/Crab-eating_fox.pdf

165. MacPherson CN, Karstad L, Stevenson P, Arundel JH. Hydatid disease in the Turkana District of Kenya. III. The significance of wild animals in the transmission of *Echinococcus granulosus*, with particular reference to Turkana and Masailand in Kenya. Ann Trop Med Parasitol 1983; 77(1): 61-73.

166. Henttonen H, Fuglei E, Gower CN, Haukisalmi V, Ims RA, Niemimaa J, et al. *Echinococcus multilocularis* on Svalbard: introduction of an intermediate host has enabled the local life-cycle. Parasitology 2001; 123: 547-552.

167. Sokolov VE, Lushchekina AA. *Procapra gutturosa*. In Mammalian Species, No. 571. American Society of Mammalogists 1997 [cited 2007 Apr 26]. Available from http://www.jstor.org/view/00763519/ap060706/06a00010/0

168. Krecek RC, Boomker J, Penzhorn BL, Scheepers L. Internal parasites of giraffes (*Giraffa camelopardalis angolensis*) from Etosha National Park, Namibia. J Wildl Dis 1990; 26(3): 395-397.

169. World Organisation for Animal Health – OIE Working Group for Wildlife Diseases. Regional report for Africa, 2005. Compiled by Bengis R [cited 2007 Apr 26]. Available from http://www.nda.agric.za/vetweb/Animal%20Disease/OIERegionalReportForAfrica2005.pdf

170. Hamir AN, Smith BB. Severe biliary hyperplasia associated with liver fluke infection in an adult alpaca. Vet Pathol 2002; 39(5): 592-594.

171. Bassano B. Sanitary problems related to Marmot-other animals cohabitation in mountain areas. In Biodiversité chez les marmottes /Biodiversity in marmots, 1996, Le Berre M., Ramousse R. & L. Le Guelte eds., 75-88.

172. Williams JF, Colli CW. Primary cystic infection with *Echinococcus granulosus* and *Taenia hydatigena* in *Meriones unguiculatus*. J Parasitol 1970; 56(3): 509-513.

173. Public Health Agency of Canada. Material Safety Data Sheet – Infectious Substances: *Echinococcus multilocularis* [cited 2007 Apr 26]. Available from http://www.phac-aspc.gc.ca/msds-ftss/msds55e.html

174. Tsukada H, Hamazaki K, Ganzorig S, Iwaki T, Konno K, Lagapa JT, et al. Potential remedy against *Echinococcus multilocularis* in wild red foxes using baits with anthelmintic distributed around fox breeding dens in Hokkaido, Japan. Parasitology 2002; 125: 119-129.

175. Romano MN, Brunetti OA, Schwabe CW, Rosen MN. Probable transmission of *Echinococcus granulosus* between deer and coyotes in California. J Wildl Dis 1974; 10: 225-227.

176. Boussinesq M, Bresson S, Liance M, Houin R. A new natural intermediate host of *Echinococcus multilocularis* in France: the muskrat (*Ondatra zibethicus L.*) [Article in French]. Ann Parasitol Hum Comp 1986; 61(4): 431-434.

177. Gonzalez del Solar R, Rau J. Chilla *Pseudalopex griseus* (Gray, 1837) [cited 2007 Apr 26]. Available from http://www.canids.org/species/Chilla.pdf

178. D'Alessandro A, Rausch RL, Morales GA, Collet S, Angel D. *Echinococcus* infections in Colombian animals. Am J Trop Med Hyg 1981; 30(6): 1263-1276.

179. Bardonnet K, Benchikh-Elfegoun MC, Bart JM, Harraga S, Hannache N, Haddad S, et al. Cystic echinococcosis in Algeria: cattle act as reservoirs of a sheep strain and may contribute to human contamination. Vet Parasitol 2003; 116(1): 35-44.

180. Varma TK, Arora BM, Malviya HC. On the occurrence of hydatid cyst in giant squirrel (*Ratufa indica*). Indian Vet J 1995; 72(12): 1305-1306.

181. Machnicka B, Dziemian E, Rocki B, Kolodziej-Sobocinska M. Detection of *Echinococcus multilocularis* antigens in faeces by ELISA. Parasit Res 2003; 91(6): 491-496.

182. Boomker J, Horak IG, de Vos V. Parasites of South African wildlife. IV. Helminths of kudu, *Tragelaphus strepsiceros*, in the Kruger National Park. Onderstepoor J Vet Res 1989; 56(2): 111-121.

183. Wobeser G. The occurrence of *Echinococcus multilocularis* (Leukart, 1863) in cats near Saskatoon, Saskatchewan. Can Vet J 1971; 12(3): 65-68.

184. Bakhvalova VN, Morozova OV, Dobrotvorskiĭ AK, Panov VV, Matveeva VA, Popova RV, Korobova SA. Involvement of the common shrew, *Sorex araneus* (Insectivora, Soricidae), in circulation of the tick-borne encephalitis virus in south-western Siberia [in Russian]. Parazitologiia 2001; 35(5): 376-385.

185. Janovsky M, Bacciarini L, Sager H, Grone A, Gottstein B. *Echinococcus multilocularis* in a European beaver from Switzerland. J Wildl Dis 2002; 38(3): 618-620.

186. Munday BL. A Serological study of some infectious diseases of Tasmanian wildlife. J Wildl Dis 1972; 8(4): 169-175.

187. World Health Organization. WHO Guidelines on tulaeremia. 2007 [cited 2007 Apr 26]. Available from http://whqlibdoc.who.int/publications/2007/9789241547376_eng.pdf

188. Morvan JM, Deubel V, Gounon P, Nakoune E, Barriere P, Murris S, et al. Identification of Ebola virus sequences present as RNA or DNA in organs of terrestrial small mammals of the Central African Republic. Microbes Infect 1999; 1(14): 1193-1201.

189. Calisher CH, Childs JE, Field HE, Holmes KV, Schountz T. Bats: Important reservoirs of emerging viruses. Clin Microbiol Rev 2006; 19(3): 531-545.

190. Klempa B; Tkachenko EA; Dzagurova TK, Yunicheva YV, Morozov V, Okulova NM, et al. Hemorrhagic fever with renal syndrome caused by 2 lineages of Dobrava hantavirus, Russia. Emerg Infect Dis 2008; 14(4): 617-625.

191. Coulibaly C, Hack R, Seidl J, Chudy M, Itter G, Plesker R. A natural asymptomatic herpes B virus infection in a colony of laboratory brown capuchin monkeys (*Cebus apella*). Laboratory Animals 2004; 38(4): 432-438.

192. Wilson RB, Holscher MA, Chang T, Hodges JR. Fatal herpesvirus simiae (B virus) infection in a patas monkey (*Erythrocebus patas*). J Vet Diagn Invest 1990; 2: 242-244.

193. Tu C, Crameri G, Kong X, Chen J, Sun Y, Yu M, et al. Antibodies to SARS coronavirus in civets. Emerg Infect Dis 2004; 10(12): 2244-2248.

194. Ward O, Wurster-Hill D. Ecological studies of Japanese raccoon dogs, *Nyctereutes procyonoides*. J Mammal 1989; 70: 330-334.

195. Loris-Conservation.org [cited 2007 Apr 27]. Available from http://www.loris-conservation.org/database/disease/index.htm#diseases

196. Pattnaik P. Kyasanur forest disease: an epidemiological view in India. Rev Med Virol 2006; 16: 151–165.

197. Peters JC. An epizootic of monkey pox at Rotterdam Zoo. Int Zoo Yearb 1966; 6: 274-275.

198. Hutin YJF, Williams RJ, Malfait P, Pebody R, Loparev VN, Ropp SL, et al. Outbreak of human monkeypox, Democratic Republic of Congo, 1996 to 1997. Emerg Infect Dis 2001; 7(3): 434-438.

199. Smits WTM, Heriyanto, Ramono WS. A New method for rehabilitation of orangutans in Indonesia. In: Nadler RD, Galdikas FM, Sheeran LK, Rosen N, eds. The Neglected ape. New York: Plenum Press. 1995. pp. 69-77.

200. Matz-Rensing K, Floto A, Becker T, Finke EJ, Seibold E, Splettstoesser WD, et al. Epizootic of tularemia in an outdoor housed group of cynomolgus monkeys (*Macaca fascicularis*). Vet Pathol 2007; 44: 327-334.

201. National Research Council of the National Academies. Occupational Health and Safety in the Care and Use of Nonhuman Primates. Washington DC: National Academies Press. 2003.

202. Yimam AE, Nonaka N, Oku Y, Kamiya M. Prevalence and intensity of *Echinococcus multilocularis* in red foxes (*Vulpes vulpes schrencki*) and raccoon dogs (*Nyctereutes procyonoides albus*) in Otaru city, Hokkaido, Japan. Jap J Vet Res 2002; 49(4): 287-296.

203. Clemente L, Fernandes TL, Barahona MJ, Bernardino R, Botelho A. Confirmation by PCR of *Coxiella burnetii* infection in animals at a zoo in Lisbon, Portugal. Vet Rec 2008; 163: 221-222.

204. Moles-Cervantes LP, Pulido-Reyes J, Banda-Ruiz VM, Luna-Alvarez MA, Galvadon-Rosas DG, Torres-Barranca JI. Serological diagnosis of leptospirosis in a giant panda (*Ailuropoda melanoleuca*) in Spanish]. Tecnica Pecuaria en Mexico 1994; 32(3): 145-149.

205. Hutson CL, Lee KN, Abel J, Carroll DS, Montgomery JM, Olson VA, et al. Monkeypox zoonotic associations: insights from laboratory evaluation of animals associated with the multi-state US outbreak. Am J Trop Med Hyg 2007; 76(4): 757-768.

206. Dierauf LA, Gulland FMD. CRC Handbook of Marine Mammal Medicine. Boca Raton: CRC Press, 2001.

207. Foster G, Jahans KL, Reid RJ, Ross HM. Isolation of *Brucella* species from cetaceans, seals and an otter. Vet Rec 1996; 138(24): 583-6.

208. Murray DL, Kapke CA, Evermann JF, Fuller TK. Infectious disease and the conservation of free-ranging large carnivores. Anim Conserv 1999; 2: 241-254.

209. International Union for Conservation of Nature. Mills MGL and Hofer H, eds. Hyaenas Status Survey and Conservation Action Plan. Gland Switzerland: 1998.

210. Nielsen K and Duncan JR. Animal brucellosis. Boca Raton: CRC Press, 1990.

211. Towner JS, Pourrut X, Albariño CG, Nkogue CN, Bird BH, Grard G, et al. Marburg virus infection detected in a common African bat. PLoS ONE 2007; 2(8): e764.

212. Food and Agriculture Organization. Avian Flu [cited 2007 Apr 29]. Available from http://www.fao.org/avianflu/en/wildlife/wild_animals.htm

213. Songserm T, Amonsin A, Jam-on R, Sae-Heng N, Pariyothorn N, Payungporn S. Fatal avian influenza A H5N1 in a dog. Emerg Infect Dis 2006; 12(11): 1744-1747.

214. US Geological Survey National Wildlife Health Center. List of species affected by H5N1 (avian influenza) [cited 2007 Apr 29]. Available from http://www.nwhc.usgs.gov/disease_information/avian_influenza/affected_species_chart.jsp

215. Prosenc K, Avsic-Zupanc T, Trilar T, Petrovec M, Poljak M. The fat dormouse *Myoxus glis* as a natural host of medically important microorganisms. Nat Croat 1997; 6(2): 253-262.

216. Gudan A, Artuković B, Cvetnić Z, Spicić S, Beck A, Hohsteter M, et al. Disseminated tuberculosis in hyrax (*Procavia capensis*) caused by *Mycobacterium africanum*. J Zoo Wildl Med 2008; 39(3): 386-391.

217. Koizumi N, Muto M, Yamamoto S, Baba Y, Kudo M, Tamae Y, et al. Investigation of reservoir animals of Leptospira in the northern part of Miyazaki Prefecture. Jpn J Infect Dis 2008; 61(6): 465-8.

218. Cornide RI, Cabrera L. [Detection of *Leptospira* antibodies in Hutia (*Mysateles* spp.)] [Article in Spanish] Miscelánea Zoológica. 1984; 22: [pages not cited]. Reference within Monografias.com: "*Leptospira interrogans*." [Cited 2009 Feb 26]. Available from http://www.monografias.com/trabajos25/leptospira-interrogans/leptospira-interrogans.shtml

219. Pioz M, Loison A, Gauthier D, Gibert P, Jullien JM, Artois M, et al. Diseases and reproductive success in a wild mammal: example in the alpine chamois. Oecologia 2008; 155(4): 691-704.

220. Maratea J, Ewalt DR, Frasca S Jr, Dunn JL, De Guise S, Szkudlarek L, et al. Evidence of *Brucella* sp. infection in marine mammals stranded along the coast of southern New England. J Zoo Wildl Med 2003; 34(3): 256-61.

221. Blasdell KR, Becker SD, Hurst J, Begon M, Bennett M. Host range and genetic diversity of arenaviruses in rodents, United Kingdom. Emerg Infect Dis 2008; 14(9): 1455-1458.

222. Normile D. Scientists puzzle over Ebola-Reston virus in pigs. Science 2009; 323(5913): 451.

223. Swanepoel R, Smit SB, Rollin PE, Formenty P, Leman PA, Kemp A, et al. Studies of reservoir hosts for Marburg virus. Emerg Infect Dis 2007; 13(12): 1847-1851.

224. Shi Z, Hu Z. A review of studies on animal reservoirs of the SARS coronavirus. Virus Res 2008; 133: 74–87.

225. Greenwood AG, Sanchez S. Serological evidence of murine pathogens in wild grey squirrels (*Sciurus carolinensis*) in North Wales. Vet Rec 2002; 150(17): 543-6.

226. Serra-Cobo J, Amengual B, Abellán C, Bourhy H. European bat lyssavirus infection in Spanish bat populations. Emerg Infect Dis 2002; 8(4): 413-20.

227. Paweska JT, Blumberg LH, Liebenberg C, Hewlett RH, Grobbelaar AA, Leman PA, et al. Fatal human infection with rabies-related Duvenhage virus, South Africa. Emerg Infect Dis 2006; 12(12): 1965-7.

228. Pfukenyi DM, Pawandiwa D, Makaya PV, Ushewokunze-Obatolu U. A retrospective study of wildlife rabies in Zimbabwe, between 1992 and 2003. Trop Anim Health Prod. 2008 Aug 30; [Epub ahead of print].

229. Li Y, Wang J, Hickey AC, Zhang Y, Li Y, Wu Y, et al. Antibodies to Nipah or Nipah-like viruses in bats, China. Emerg Infect Dis 2008; 14(12): 1974–1976.

230. Lednicky JA. Hantaviruses: A short review. Arch Pathol Lab Med 2002; 127(1): 30-35.

231. McNamara T, Linn M, Calle P, Cook R, Karesh W, Raphael B. Leptospirosis: an under-reported disease in zoo animals? Verh ber Erkrg Zootiere 1999; 39: 185-188.

232. McCall BJ, Epstein JH, Neill AS, Heel K, Field H, Barrett J, et al. Potential human exposure to Australian bat lyssavirus, Queensland, 1996-1999. Emerg Infect Dis 2000; 6(3): 259-264.

233. International Committee on Taxonomy of Viruses. The universal database of the International Committee on Taxonomy of Viruses [cited 2009 September 19]. Available from http://www.ncbi.nlm.nih.gov/ICTVdb

234. Cousins DV, Bastida R, Cataldi A, Quse V, Redrobe S, Dow S, et al. Tuberculosis in seals caused by a novel member of the *Mycobacterium tuberculosis* complex: *Mycobacterium pinnipedii* sp. nov. Int J Syst Evol Micr 2003;53:1305–14.
